# Synthesis and antioxidant activities of benzylic bromophenols inclusive of natural products

**DOI:** 10.55730/1300-0527.3447

**Published:** 2022-05-06

**Authors:** Çetin BAYRAK, Eda Mehtap ÜÇ, Mohsen REZAEI, İhami GÜLÇİN, Abdullah MENZEK

**Affiliations:** 1 Department of Chemistry, Faculty of Science, Atatürk University, Erzurum, Turkey; 2 Doğubayazıt Ahmed-i Hani Vocational School, Ağrı İbrahim Çeçen University, Ağrı, Turkey

**Keywords:** Antioxidant activity, bromination, bromophenol, radical scavenging, natural product, benzylic acid

## Abstract

The synthesis of natural products 2-(2,3-dibromo-4,5-dihydroxyphenyl)acetic acid (**1**) and 2-(2,6-dibromo-3,5-dihydroxyphenyl)acetic acid (**2**) and as well as their derivatives **25** and **26** were carried out by substitution, hydrolysis and demethylation reactions of the corresponding four benzyl bromides. The antioxidant potentials of benzylic acid-derived bromophenols were, for the first time, appraised by several outstanding bioanalytical methods. Besides these, we estimated the antioxidant effects which were studied using the methods of DPPH·, ABTS^•+^ scavenging activities, ferric (Fe^3+^) and cupric (Cu^2+^) ions and Fe^3+^-TPTZ reducing capacities. Benzylic acid-derived bromophenols were found as effective DPPH^•^, and ABTS^•+^ scavengers. The potential antioxidant activities of bromophenol derivatives **1, 2** and **17–28** were compared to standard antioxidants including BHA, BHT, α-Tocopherol, and Trolox, which is a water-soluble analog of vitamin E. We expect that this innovative work will direct future studies exploring the antioxidant properties of food, medicinal, and industrial applications.

## 1. Introduction

Natural bromophenols are commonly found in marine life [1]. Many biological functions including antioxidant [2–5], antimicrobial [6,7], and carbonic anhydrase (CA) inhibition have been investigated for them and their derivatives [8–14].

**Figure 1:**
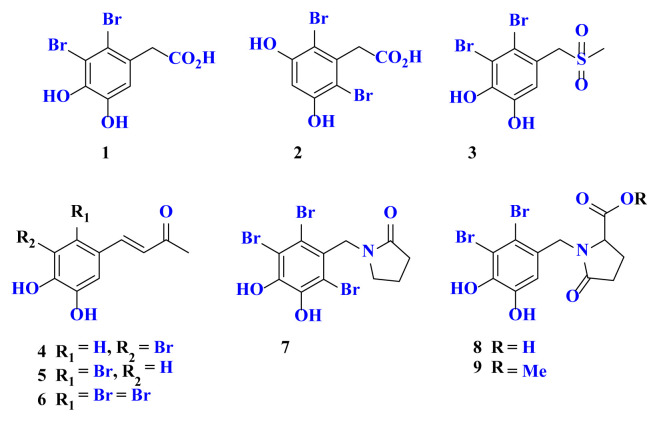
Some natural bromophenols.

Bromophenols **1–9** seen in Figure 1 are natural bromophenols [2,15–18]. Natural bromophenols 1 and 2 were isolated from the *Halopitys* incurvus algae of the Rhodomelaceae [15] and the red algae of *Rhodomelaceae confervoides* [16], respectively. From these natural bromophenols **1–9**, we reported the first synthesis as well as various biological activities such as carbonic anhydrase of **3–9** in our previous studies (Figure 1) [5,11,13].

Oxidative stress is associated with an imbalance between the antioxidants and the reactive oxygen species (ROS) in the body. This situation is known to cause the development of many chronic diseases [19–21]. The excess formation of free radicals and ROS cause degenerative damage to vital cellular molecules including carbohydrates, proteins, lipids, and nucleic acids [22–24]. As a result, oxidative stress and ROS are known as important environmental factors leading to many chronic diseases such as cancer, cardiovascular diseases, immunodeficiency syndrome, obesity, age-related pathologies, arteriosclerosis, and diabetes mellitus [25,26]. Even at low concentrations, antioxidants are quite effective at counteracting the detrimental effects of both oxidative stress and ROS. Phenolic compounds obtained from natural sources, especially those found in plants, exhibit a wide range of biological activity. They have also been intensely investigated due to their possible antioxidant and biological abilities [27–29]. Antioxidants are chemicals that are preferentially oxidized, preventing or completely inhibiting the oxidation of other oxidizable compounds which, once oxidized, may be hazardous effects on food or pharmacological products [30,31]. The most common synthetic antioxidants approved for use in food today are butylated hydroxyanisole (BHA) and butylated hydroxytoluene (BHT), tertiary-butylhydroquinone, and propyl gallate [32,33]. On account of the serious safety concerns regarding petrochemical antioxidants, there is an increasing demand for natural or naturally derived antioxidants due to their positive effects on human health [34]. In this way, natural antioxidants and their derivatives play an important role in living systems and in human health. Moreover, antioxidants also have an important role as therapeutic effects in many chronic diseases [35].

The synthesis of the bromophenols **1** and **2** is desirable because they are natural products that are potentially vital compounds for many biological processes. For this reason, the synthesis of compounds **1** and **2**, as well as their derivatives, was performed and the resulting compounds were investigated for their antioxidant properties using various bioanalytical methods.

## 2. Result and discussion

### 2.1. Chemistry

**Scheme 1.**
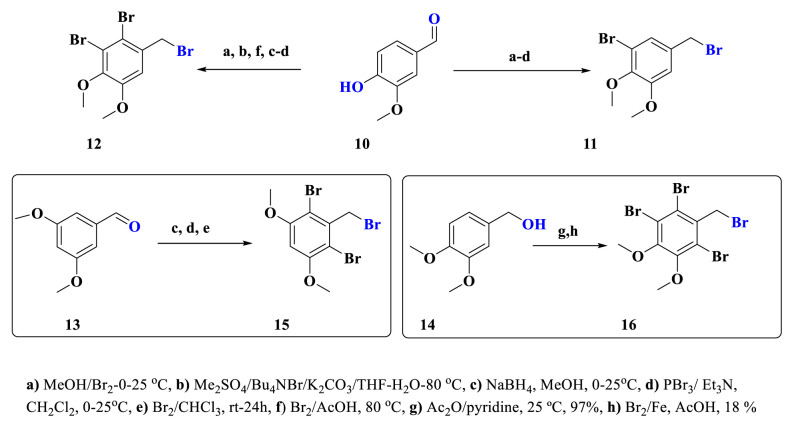
Synthesis of benzyl bromides **11**, **12, 15**, and **16**.

Natural bromophenols **1** and **2** both contain a benzylic acid group, as well as two Br and two ^−^OH groups at varied positions around the ring (Figure 1). To obtain these products and derivatives, vanillin (**10**), 3,5-dimethoxybenzaldehyde (**13**) and (3,4-dimethoxyphenyl)methanol (**14**) were chosen as starting compounds. Benzyl bromides **11**, **12, 15**, and **16** are already known and were synthesized by established methods in the literature (Scheme 1) [36–40].

**Scheme 2.**
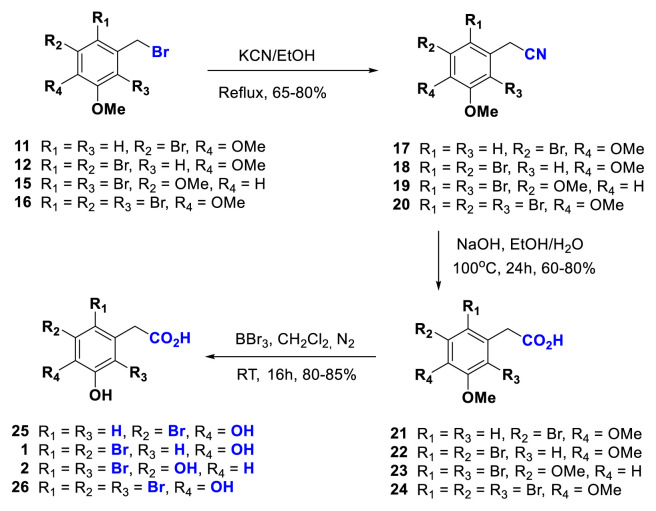
As bromophenols, synthesis of 2-phenylacetic acid derivatives from the corresponding bromides via their nitriles.

According to the known procedure [41], substitution nitrile substituted compounds **17–20** were obtained from their corresponding bromides **11**, **12**, **15**, and **16** (Scheme 2). As expected, in ^1^H-NMR spectra, an upfield shift is shown in the benzylic hydrogens of compounds **17–20** owing to the decreased electron-withdrawing nature of the nitrile group. Also, all data belonging to them suggested structures (Scheme 2).

The nitrile groups in the **17–20** were then hydrolyzed under basic conditions (NaOH in the EtOH/H_2_O) (Scheme 2). The obtained compounds are carboxylic acids (**21–24**), as is evidenced by the appearance of carbonyl groups ranging between 170–177 ppm in their ^13^C-NMR spectra. Furthermore, the appearance of peaks around 10.5 ppm in the ^1^H-NMR spectra of compounds **21** and **24** is indicative of the conversion of these nitrile groups into the corresponding carboxylic acid groups (Scheme 2).

Bromophenols are important compounds because of biologically active [1–16]. Bromophenols 1 and 2 are more important because they are both biologically active and natural products [15,16]. The compounds 22 and 23 are precursor compounds for natural bromophenol compounds 1 and 2.

Finally, the synthesis of bromophenols 1, 2 was performed by treating 22 and 23 with BBr3. Similarly, the reactions of the compounds 21 and 24 with BBr3 yielded bromophenol derivatives 25 and 26 (Scheme 2).

**Scheme 3.**
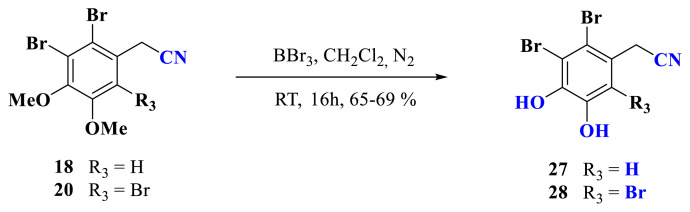
Synthesis of bromophenol derivatives **27** and **28** from the compounds **18** and **20**.

In addition to the benzylic acid-containing bromophenols **1**, **2**, **25**, and **26**, benzyl nitrile containing bromophenols **27** and **28** were also sought after due to their important biologic activities. Therefore, their synthesis was carried out by treating compounds **18** and **20** with BBr_3_ (Scheme 3). NMR data of bromophenol derivatives **1**, **2**, and **25–28** are consistent with their structures.

### 2.2. Biological activities

#### 2.2.1. Reducing power results

**Figure 2:**
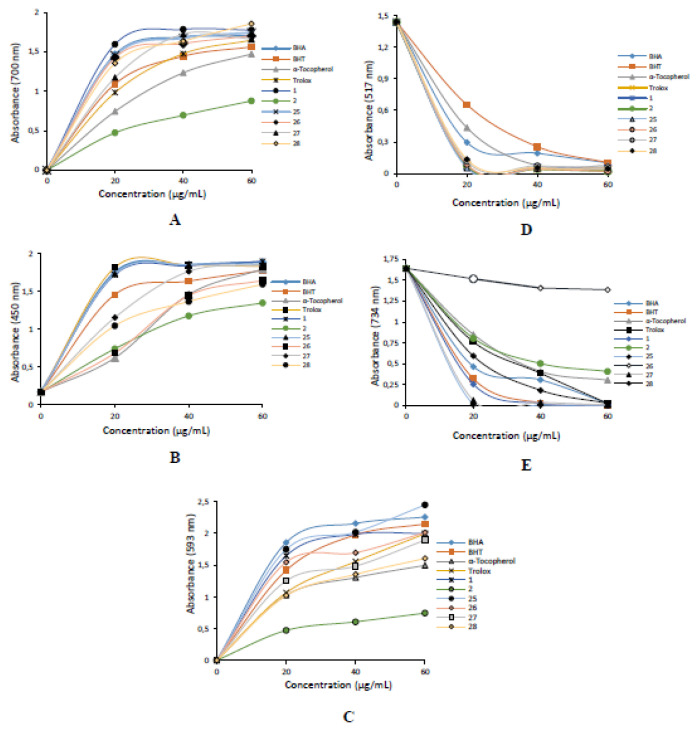
Different antioxidant assay for bromophenol derivatives **1, 2**, and **17–28**: **A**. Fe^3+^ reducing method, **B**. Cu^2+^ reducing method, **C**. Fe^3+^-TPTZ reducing method, **D**. DPPH· scavenging method, **E**. ABTS^•+^ scavenging method.

Reducing power can donate the capacity of bioactive biological compounds that act as reductants and inactivate ROS and oxidant agents [42]. Fe^3+^ reduction ability assay measures the reducing potential of the compounds. Fe^3+^ ions addition to bromophenol derivatives **1, 2**, and **17–28** occur in blue colored complex of Fe_4_[Fe(CN-)_6_]_3_. This complex demonstrated absorbance at 700 nm [43,44]. As a result of the complex, the yellow color of samples varies from green to blue according to the effectiveness of test compounds [45]. In this sense, bromophenol derivatives **1, 2** and **17–28** demonstrated potent and efficient reducing ability by using the Fe[Fe(CN-)_6_]_3_, Cu^2+^ and Fe^3+^-TPTZ reduction abilities. For measuring of reduction ability of bromophenol derivatives **1, 2**, and **17–28**, Fe^3+^-Fe^2+^ transformation was realized according to Oyaizu’s method [46]. As given in Table 1 and Figure 1A, bromophenol derivatives **1, 2**, and **17–28**, demonstrated efficient Fe^3+^ reducing capability. Since the reducing potencies and radical scavenging capacities of bromophenol derivatives **17–24** are relatively lower due to the structure-activity relationship, they will not be considered much in the discussion part. The other bromophenol derivatives **17–24** demonstrated moderate Fe^3+^ reducing ability ranging from 0.253 ± 0.004–0.463 ± 0.010 when compared to standards. Generally, the antioxidant properties of the test sample are compared with standard antioxidants. Many compounds are used as standards for this purpose. In addition, the standard selection and criteria are also related to the stability, price, and solubility of the standard antioxidant in the solvent environment [47]. The increased absorbance shows the complex formation and increased enhanced reducing effect (Figure 2A). The results clearly show that bromophenol derivatives **1, 2**, and **17–28** have strong Fe^3+^ reducing effects with e^−^ donating properties for neutralizing free radicals and ROS. They can apply in biochemical and biological systems to reduce oxidative stress or damage.

Aside Fe^3+^-TPTZ reduction abilities, Cu^2+^ reducing of bromophenol derivatives **1, 2**, and **17–28** are given in Table 1 and Figure 2B. A positive correlation was observed between the Cu^2+^ reducing and bromophenol derivatives **1, 2**, and **17–28** as concentration-dependently (20–60 μg/mL). At the concentration of 60 μg/mL, Cu^2+^ reducing capability of bromophenol derivatives **1, 2**, and **17–28** and standards were declined as following orders (Table 1 and Figure 2B): **1** (1.896 ± 0.002, r^2^: 0.6828) > BHA (1.864 ± 0.015, r^2^: 0.9854) ≈ **25** (1.853 ± 0.003, r^2^: 0.6573) > Trolox (1.829 ± 0.004, r^2^: 0.6061) > α-Tocopherol (1.795 ± 0.003, r^2^: 0.9747) > BHT (1.744 ± 0.003, r^2^: 0.7642) > **26** (1.642 ± 0.002, r^2^: 0.9589) > **27** (1.538 ± 0.110, r^2^: 0.9015) > **28** (1.609 ± 0.12, r^2^: 0.9589) > **2** (1.345 ± 0.002, r^2^: 0.9507). The Fe^3+^ reducing effects of bromophenol derivatives **1, 2**, and **17–28** and standards declined as following orders: **28** (1.856 ± 0.002, r^2^: 0.8206) > **1** (1.775 ± 0.003, r^2^: 0.8992) > BHA (1.744 ± 0.004, r^2^: 0.7114) ≈ **25** (1.743 ± 0.002, r^2^: 0.6555) > **26** (1.704 ± 0.002, r^2^: 0.7336) > **27** (1.663 ± 0.003, r^2^: 0.8012) > Trolox (1.648 ± 0.007, r^2^: 0.8992) > BHT (1.563 ± 0.003, r^2^: 0.8358) > α-Tocopherol (1.473 ± 0.003, r^2^: 0.9499) > **2** (0.877 ± 0.020, r^2^: 0.9463). The other bromophenol derivatives **17–24** exhibited weaker Cu^2+^ reducing capability between in 0.253 ± 0.004–0.445 ± 0.001 when compared to standard reducing agents. The CUPRAC test had low-cost and is a rapid, stable and selective assay for different antioxidants, regardless of chemical type and hydrophobicity [48].

Aside Fe^3+^ and Cu^2+^ reduction properties of bromophenol derivatives **1, 2**, and **17–28**, they had powerful reducing potentials in FRAP assay (Figure 1C and Table 2). Reducing ability of bromophenol derivatives **1, 2**, and **17–28** was found to be in descending order of **25** (2.455 ± 0.004, r^2^: 0.8362) > BHA (2.254 ± 0.004, r^2^: 0.7435) > BHT (2.146 ± 0.002, r^2^: 0.8599) > **26** (2.016 ± 0.002, r^2^: 0.7929) > **1** (1.996 ± 0.002, r^2^: 0.7367) ≈ Trolox (1.993 ± 0.004, r^2^: 0.9494) > **27** (1.894 ± 0.002, r^2^: 0.8755) > **28** (1.605 ± 0.001, r^2^: 0.8889) > α-Tocopherol (1.497 ± 0.002, r^2^: 0.8531) > **2** (0.744 ± 0.002, r^2^: 0.8929). The other bromophenol derivatives **17–24** showed relatively weaker Fe^3+^-TPTZ reducing ability between in 0.304 ± 0.004–0.667 ± 0.002 when compared to standard reducing compounds. As mentioned in prior reduction assay, high reducing absorbance shows high reducing ability of the complex. The FRAP method is realized in an acidic medium to protect iron ions solubility [49].

#### 2.2.2. Radicals scavenging results

DPPH· and ABTS^˙+^ scavenging assays are among the most convenient spectrophotometric scavenging methods. Both assays were used to determine the antioxidant abilities and radical scavenging capacities of plants, foods, and beverages [50]. The percent inhibition depends on the concentrations of oxidizers such as antioxidants and radicals, the ratios of solvent and reagent used, incubation time, temperature, and also the presence of hydrogen, metal, and water in the antioxidant test systems [51]. On the other hand, IC_50_ is called the effective concentration that causes 50% removal of oxidants such as radicals in antioxidant studies. It is often used to assess the antioxidant activity [47]. For DPPH radical scavenging were found to be in following order: BHT (IC_50_: 4.12 μg/mL, r^2^: 0.9690) > **25** (IC_50_: 4.27 μg/mL, r^2^: 0.9016) > **1** (IC_50_: 6.41 μg/mL, r^2^: 0.9961) > **27** (IC_50_: 6.86 μg/mL, r^2^: 0.9018) > **28** (IC_50_: 10.66 μg/mL, r^2^: 0.9652) > BHA (IC_50_: 11.17 μg/mL, r^2^: 0.9030) > Trolox (IC_50_: 11.75 μg/mL, r^2^: 0.8513) > α-Tocopherol (IC_50_: 23.89 μg/mL, r^2^: 0.9732) > **2** (IC_50_: 30.13 μg/mL, r^2^: 0.9457) > **26** (IC_50_: 231.00 μg/mL, r^2^: 0.9652). On the other hand, the other bromophenol derivatives **17–24** demonstrated relatively weak DPPH radical scavenging ability and found IC_50_ values between 17.32–346.50 μg/mL when compared to standard radical scavengers. A lower IC_50_ value demonstrates a higher DPPH· scavenging ability (Table 2 and Figure 1D). In another study, DPPH radical scavenging activity of nineteen bromophenols from *Rhodomela confervoides* was realized. Among these bromophenols, bromophenols **1** was also studied. It was shown that bromophenols **1** had IC_50_ value of 19.84 μM for DPPH radical scavenging activity. Also, it was demonstrated that the metabolites with *ortho*-dihydroxy groups on the aromatic ring generally display higher activity than the compounds having a single free hydroxyl group on the ring [16,52]. The bromophenol derivatives **1, 2**, and **17–28** exhibited effective ABTS^˙+^ removing ability. As given in Table 2 and Figure 1E, bromophenol derivatives **1, 2**, and **17–28** effectively scavenged ABTS radicals as concentration-dependently (20–60 μg/mL, p < 0.001). EC_50_ values of bromophenol derivatives **1, 2**, and **17–28** in ABTS^˙+^ scavenging assay were found to be in descending order of **25** (IC_50_: 9.36 μg/mL, r^2^: 0.6059) > Trolox (IC_50_: 9.36 μg/mL, r^2^: 0.6119) ≈ **26** (IC_50_: 9.49 μg/mL, r^2^: 0.8680) > **1** (IC_50_: 9.90 μg/mL, r^2^: 0.6119) > **28** (IC_50_: 10.19 μg/mL, r^2^: 0.6496) > **27** (IC_50_: 10.28 μg/mL, r^2^: 0.6654) > **2** (IC_50_: 10.66 μg/mL, r^2^: 0.8511) > BHA (IC_50_: 14.74 μg/mL, r^2^: 0.7129) > BHT (IC_50_: 15.75 μg/mL, r^2^: 0.9986) > α-Tocopherol (IC_50_: 12.15 μg/mL, r^2^: 0.7950). On the other hand, the other bromophenol derivatives **17–24** demonstrated relatively weak DPPH radical scavenging ability and found IC_50_ values when compared to standard radical scavengers. As DPPH in radical scavenging, a lower IC_50_ value shows higher ABTS^˙+^ scavenging ability. In a previous study, it was shown that 2.87 mM TEAC ABTS^˙+^ scavenging ability. In this study, the isolated nineteen bromophenols were found effective radical scavenging potential against ABTS cation radicals [16]. Already, it is well-known that ABTS radical scavenging properties of antioxidants can attribute H-donating effect [53].

## 3. Conclusion

As a result, after bromides **11**, **12**, **15**, and **16** were synthesized, the acids including natural products were obtained via the corresponding nitriles **17–20**. Chronic diseases, mutagenesis, DNA damage, carcinogenesis, and inhibition of pathogenic bacterial growth are generally associated with the scavenging of ROS and free radical propagation in living systems. Antioxidant activity is used as an effective and common parameter for medicinal bioactive components and newly synthesized biologically important molecules. For this reason, natural products **1** and **2**, including their derivatives **25** and **26** were synthesized through the conversion of benzyl bromides **11–16** into the corresponding nitriles **17–20** followed by base catalyzed hydrolysis and demethylation. In addition to these products, benzyl nitriles **27** and **28** were also synthesized through direct demethylation of **18** and **20**. Once these bromophenol derivatives were obtained **1, 2**, and **17–28** they were assessed for their antioxidant ability and compared with established antioxidants including, BHA, α-tocopherol, BHT, and Trolox.

## 4. Experimental section

### 4.1. General procedures

Chemicals (including solvents) used in the experiments and data of the synthesized compounds (such as NMR and HRMS) were performed as previously stated. [14,54]. Reagent benzyl bromides **11**, **12**, **15**, and **16** were prepared in known ways in the literature [36–40].

### 4.2. Synthesis

#### 4.2.1. Synthesis of 2-(3-bromo-4,5-dimethoxyphenyl)acetonitrile (17): Standard procedure for the substitution reaction with KCN

KCN (315 mg, 4.84 mmol) was added to the stirred solution of bromide **11** (500 mg, 1.61 mmol) in EtOH (20 mL). After refluxing of the solution at 80 °C for 16 h, termination of the reaction, removal of the solvent under vacuum and extraction of reside with EtOAc (2 × 20 mL) were performed, respectively. Then, combination and drying over Na_2_SO_4_ of organic phases and evaporation of the solvent under vacuum were carried out. After purification of the residue by column chromatography on silica gel (10 g) using EtOAc:hexane (1:9) eluent, the product **17** (270 mg, 65%, white solid) was obtained. Mp: 76–77°C; ^1^H NMR (400 MHz, CDCl_3_): 7.08 (s, 1H, ArH), 6.80 (s, 1H, ArH), 3.88 (s, OMe, 3H), 3.84 (s, OMe, 3H), 3.68 (s, CH_2_, 2H); ^13^C NMR (100 MHz, CDCl_3_): 154.26, 146.58, 127.04, 124.31 (CH), 118.31, 117.61 (C). 111.54 (CH), 60.84 (OMe), 56.43 (OMe), 23.28 (CH_2_); IR (CH_2_Cl_2_, cm^−1^): 2939, 2251, 1570, 1491, 1416, 1274, 1141, 1046 cm^−1^; HRMS (m:z): calcd. for [C_10_H_10_^79^BrNO_2_]^+^: 254.9895; found 254.9896, R_f_: 0.51; EtOAc:hexane: (3:7).

#### 4.2.2. Synthesis of 2-(2,3-dibromo-4,5-dimethoxyphenyl)acetonitrile (18)

Using standard procedure written in 4.2.1, the product **18** (280 mg, 65%, yellow solid) was obtained. Mp: 121–122 °C; ^1^H NMR (400 MHz, CDCl_3_): 7.08 (s, 1H, ArH), 3.91 (s, OCH_3_, 3H), 3.87 (s, CH_2_, 2H), 3.86 (s, OCH_3_, 3H); ^13^C NMR (100 MHz, CDCl_3_): 153.09, 147.83, 127.10, 122.78, 117.00, 117.06, 112.51 (CH), 60.83 (OCH_3_), 56.60 (OCH_3_), 26.72 (CH_2_); IR (CH_2_Cl_2_, cm^−1^): 2930, 2246, 1652, 1477, 1412, 1378, 1322, 1265, 1210, 1058, 1002; HRMS (m:z): calcd. for [C_10_H_9_^79^Br_2_NO_2_]^+^: 332.9000; found 332.9003; R_f_: 0.5; EtOAc:hexane: (15:85)

#### 4.2.3. Synthesis of 2-(2,6-dibromo-3,5-dimethoxyphenyl)acetonitrile (19)

Using standard procedure written in 4.2.1, the product **19** (650 mg, 75%, yellow solid) was obtained. Mp: 198–199 °C; ^1^H NMR (400 MHz, CDCl_3_): 6.53 (s, ArH, 1H), 4.18 (s, 2H), 3.92 (s, OCH_3_, 6H); ^13^C NMR (100 MHz, CDCl_3_): 156.48, 131.62, 115.76, 105.50, 96.96 (CH), 56.73 (2 OCH_3_), 26.25 (CH_2_); IR (CH_2_Cl_2_, cm^−1^): 2935, 2246, 1574, 1453, 1339, 1220, 1080, 1068 cm^−1^; HRMS (m:z): calcd. for: [C_10_H_9_^79^Br_2_NO_2_]^+^: 332.9000; found 332.9001; R_f_: 0.46; EtOAc:hexane: (3:7).

#### 4.2.4. Synthesis of 2-(2,3,6-tribromo-4,5-dimethoxyphenyl)acetonitrile (20)

Using standard procedure written in 4.2.1, the product **20** (530.00 mg, 80%, white solid) was obtained. Mp: 161–162°C; ^1^H NMR (400 MHz, CDCl_3_): 4.22 (s, CH_2_, 2H), 3.92 (s, OCH_3_, 3H), 3.91 (s, OCH_3_, 3H); ^13^C NMR (100 MHz, CDCl_3_): 152.60, 151.28, 127.88, 122.88, 122.45, 120.76, 115.63, 61.24 (OCH_3_), 61.20 (OCH_3_), 28.22 (CH_2_); IR (CH_2_Cl_2_, cm^−1^): 2935, 2246, 1372, 1092, 1047, 1008 cm^−1^; HRMS (m:z): calcd. for: [C_10_H_8_^79^Br_3_NO_2_]^+^: 410.8105; found: 410.8108; R_f_: 0.74; EtOAc:hexane: (3:7).

#### 4.2.5. Synthesis of 2-(3-bromo-4,5-dimethoxyphenyl)acetic acid (21): Standard procedure for the hydrolysis reaction

A solution of **17** (500 mg, 1.95 mmol) in EtOH (12 mL) and H_2_O (8 mL) was added to NaOH (780 mg, 19.52 mmol). After the reaction was allowed to stir for 24 h at 100 °C and was cooled to RT, removal of the solvent under vacuum, and then acidification of the reaction mixture with addition of cold HCl solution (1.0 M, 0 °C) until pH of the solution adjusted to 2.0 were performed. Respectively, extraction of the mixture with EtOAc (2 × 20 mL), combination and drying over Na_2_SO_4_ of organic phases and evaporation of the under vacuum, removal of the solvent under vacuum, and the product **21** (430 mg, 80%, yellow solid) was obtained. Mp: 106–107 °C; ^1^H NMR (400 MHz, CDCl_3_): 10.68 (s, CO_2_H, 1H), 7.05 (s, ArH, 1H), 6.77 (s, ArH, 1H), 3.85 (s, OCH_3_, 3H), 3.83 (s, OCH_3_, 3H), 3.56 (s, CH_2_, 2H); ^13^C NMR (100 MHz, CDCl_3_): 177.63 (CO), 153.83 (C), 145.97 (C), 130.41 (C), 125.72 (CH), 117.85 (C), 113.09 (CH), 60.80 (OCH_3_), 56.33 (OCH_3_), 40.65 (CH_2_); IR (CH_2_Cl_2_, cm^−1^): 3446, 1646, 1569, 1490, 1273, 1142, 1046 cm^−1^; HRMS (m:z): calcd. for: [C_10_H_11_^79^BrO_4_]^+^: 273.9841 found 273.9842, R_f_: 0.23; EtOAc:hexane: (3/7).

#### 4.2.6. Synthesis of 2-(2,3-dibromo-4,5-dimethoxyphenyl)acetic acid (22)

Using standard procedure written in 4.2.5, the product **22** (420 mg, 80%, yellow solid) was obtained. Mp: 140–141°C; ^1^H-NMR (400 MHz, CDCl_3_): 11.40-10.00 (m, COOH), 6.84 (s, ArH, 1H), 3.86 (s, CH_2_, 2H), 3.86 (s, OCH_3_, 3H), 3.84 (s, OCH_3_, 3H); ^13^C-NMR (100 MHz, CDCl_3_): 176.68 (CO), 152.69 (C), 147.29 (C), 130.91(C), 122.05 (C), 118.51 (C), 114.40 (CH), 60.74 (OCH_3_), 56.45 (OCH_3_), 43,26 (CH_2_); IR (CH_2_Cl_2_, cm^−1^): 3447, 1634, 1472, 1424, 1381, 1310, 1263, 1202, 1163, 1060, 1004; HRMS (m:z): calcd. for: [C_10_H_10_^79^Br_2_O_4_]^+^: 351.8945, found 351.8955; R_f_: 0.17, EtOAc:hexane: (3:7).

#### 4.2.7. Synthesis of 2-(2,6-dibromo-3,5-dimethoxyphenyl)acetic acid (23)

Using standard procedure written in 4.2.5, the product **23** (495 mg, 78%, white solid) was obtained. Mp: 235–236 °C (231–232 °C) [55]; ^1^H-NMR (400 MHz, acetone-d_6_): 6.71 (s, ArH, 1H) 4.02 (s, CH_2_, 2H), 3.82 (s, OCH_3_, 6H); ^13^C-NMR (100 MHz, acetone-d_6_): 170.29 (CO), 157.11 (2 C), 136.82 (C), 106.52 (2 C), 97.46 (CH), 57.04 (2 OCH_3_), 42.93 (CH_2_); IR (CH_2_Cl_2_, cm^−1^): 3330, 1702,1574, 1427, 1330, 1217, 1096 cm^−1^; HRMS (m:z): calcd. for: [C_10_H_10_^79^Br_2_O_4_]^+^: 351.8946; found: 351.8951; R_f_: 0.45; MeOH:CH_2_Cl_2_:(5:95).

#### 4.2.8. Synthesis of 2-(2,3,6-tribromo-4,5-dimethoxyphenyl)acetic acid (24)

Using standard procedure written in 4.2.5, the product **24** (250 mg, 60%, white solid) was obtained. Mp: 162–163 °C; ^1^H-NMR (400 MHz, CDCl_3_): 10.80–10.30 (m, CO_2_H, 1H), 4.27 (s, CH_2_, 2H), 3.90 (s, OCH_3_, 3H), 3.90 (s, OCH_3_, 3H); ^13^C-NMR (100 MHz, CDCl_3_): 175.62 (CO), 151.43 (C), 150.65 (C), 131.37 (C), 123.29 (C), 121.55 (C), 121.21 (C), 60.89 (OCH_3_), 60.86 (OCH_3_), 44.10 (CH_2_); IR (CH_2_Cl_2_, cm^−1^): 3444, 2935, 1703, 1651, 1395, 1285, 1093, 1010; HRMS (m/z): calcd. for: [C_10_H_9_^79^Br_3_O_4_]^+^: 429.8051, found 429.8057, R_f_: 0.30; EtOAc/Hexane: (3/7).

#### 4.2.9. Synthesis of 2-(3-bromo-4,5-dihydroxyphenyl)acetic acid (25): Standard procedure for the demethylation reaction with BBr_3_

A solution of BBr_3_ (728 mg, 2.91 mmol) in CH_2_Cl_2_ (10 mL) was added to a stirring solution of the compound **21** (400 mg, 1.45 mmol) CH_2_Cl_2_ (5 mL) under N_2_ (g) at RT, and then the mixture was stirred at the same condition for 16 h. Termination of the reaction mixture and then slow addition of H_2_O (3 mL) over 15 min at 0 °C, removal of the solvent under vacuum, the addition of H_2_O (15 mL) again and extraction of the mixture with EtOAc (2 × 25 mL) were done, respectively. After combination and drying over Na_2_SO_4_ of organic phases and evaporation of the solvent under vacuum were performed, the bromophenol **25** (290 mg 85%, brown solid) was obtained.

Mp: 166–167°C; ^1^H-NMR (400 MHz, acetone-d_6_): 8.70–8.50 (m, OH, 1H), 8.00–7.85 (m, OH, 1H), 6.96 (d, *J* = 2.0 Hz, ArH, 1H)), 6.83 (d, *J* = 2.0 Hz, ArH, 1H)), 3.48 (s, CH_2_, 2H); ^13^C-NMR (100 MHz, acetone-d_6_): 172.24 (CO), 145.79 (C), 142.02 (C), 127.69 (C), 124.39 (CH), 115.90 (CH), 109.13 (C), 39.48 (CH_2_); IR (CH_2_Cl_2_): 3523, 3309, 3204, 1702, 1496, 1434, 1289, 1093 cm^−1^; R_f_: 0.25, MeOH:CH_2_Cl_2_: (5:95). HRMS (m:z): calcd. for: [C_8_H_7_^79^BrO_4_-H]^+^: 244.9449; found 244.9454;

#### 4.2.10. Synthesis of the natural product 2-(2,3-dibromo-4,5-dihydroxyphenyl)acetic acid (1)

Using standard procedure written in 4.2.9, the natural product **1** (310 mg, 85%, brown solid) purified from EtOAc:hexane was obtained. Mp: 157–158 °C, (156–157 °C) [15]; ^1^H-NMR (400 MHz, acetone-d_6_): 6.97 (s, ArH, 1H), 3.75 (s, CH_2_, 2H); ^13^C-NMR (100 MHz, acetone-d_6_): 170.94 (CO), 144.59 (C), 127.86 (C), 117.36 (C), 117.24 (CH), 116.57 (C), 112.77 (C), 42.01 (CH_2_); IR (CH_2_Cl_2_): 3275, 1471, 1403, 1277, 1217, 1192, 1066, 1023; R_f_: 0.40, MeOH:CH_2_Cl_2_: (15:85). HRMS (m:z): calcd. for: [C_8_H_7_^79^Br^81^BrO_4_-H]^+^: 324.8534; found 324.8549.

#### 4.2.11. Synthesis of 2-(2,6-dibromo-3,5-dihydroxyphenyl)acetic acid (2)

Using standard procedure written in 4.2.9, the natural product **2** (148 mg, 80%, brown solid) was obtained. Mp: 151–152 °C (191 °C) [16]; ^1^H-NMR (400 MHz, acetone-d_6_): 9.10-8.65 (s, OH, 2H), 6.60 (s, ArH, 2H), 3.98 (s, CH_2_, 2H); ^13^C-NMR (100 MHz, acetone-d_6_): 170.38 (CO), 154.70 (2 C), 136.65 (CH), 104.66 (2 C), 103.28 (C), 42.99 (CH_2_); IR (CH_2_Cl_2_): 3616, 3445, 3199, 1706, 1432, 1200, 1093 cm^−1^; R_f_: 0.42; MeOH:CH_2_Cl_2_: (15:85); HRMS (m:z) calcd for [C_8_H_7_^79^Br_2_O_4_ - H]^+^: 322.85546; found: 322.85543.

#### 4.2.12. Synthesis of 2-(2,3,6-tribromo-4,5-dihydroxyphenyl)acetic acid (26)

Using standard procedure written in 4.2.9, the bromophenol **26** (230 mg, 82%, white solid) was obtained. Mp: 190–191 °C; ^1^H-NMR (400 MHz, acetone-d_6_): 7.00–6.60 (m, OH, 3H), 4.16 (s, CH_2_, 2H); ^13^C-NMR (100 MHz, acetone-d_6_): 170.76 (CO), 144.51 (C), 144.01 (C), 128.49 (C), 118.31 (C), 113.99 (C), 113.57 (C), 44.22 (CH_2_); IR (CH_2_Cl_2_): 3525, 3305, 3212, 1652, 1401, 1197, 1093 cm^−1^; R_f_: 0.17, MeOH:CH_2_Cl_2_: (15:85); HRMS (APCI–TOF) (m:z) calcd for [C_8_H_4_N^79^Br_2_^81^BrO_2_ - H]^−^: 402.76392; found: 402.76672.

#### 4.2.13. Synthesis of 2-(2,3-dibromo-4,5-dihydroxyphenyl)acetonitrile (27)

Using standard procedure written in 4.2.9, the bromophenol **27** (95.0 mg, 69%, brown solid) was obtained. Mp 158–159 °C; ^1^H-NMR (400 MHz, acetone-d_6_): 9.18 (bs, OH, 1H), 8.60 (bs, OH, 1H), 7.14 (s, ArH, 1H), 3.95 (CH_2_, 2H); ^13^C-NMR (100 MHz, acetone-d_6_): 145.27 (CN), 144.67 (C), 123.52 (C), 117.62 (C), 115.71 (CH), 115.47 (C), 113.67 (C), 25.32 (CH_2_); IR (CH_2_Cl_2_): 3517, 3445, 3354, 1647, 1628, 1413, 1093; HRMS (m:z) calcd for [C_8_H_5_^79^Br^81^BrNO_2_ + H_2_O + H]^+^: 325.8850; found: 325.87671.

#### 4.2.14. Synthesis of 2-(2,3,6-tribromo-4,5-dihydroxyphenyl)acetonitrile (28)

Using standard procedure written in 4.2.9, the bromophenol **28** (120.0 mg, 65%, brown solid) was obtained. M.p: 193–194 °C; ^1^H-NMR (400 MHz, acetone-d_6_): 4.15 (s, CH_2_, 2H); ^13^C-NMR (100 MHz, acetone-d_6_): 145.50, 144.43, 124.28, 117.61, 116.88, 114.04, 112.84, 28.06 (CH_2_); IR (CH_2_Cl_2_): 3521, 3312, 2324,1404, 1092 cm^−1^; R_f_: 0.42, MeOH:CH_2_Cl_2_: (5:95); HRMS (APCI – TOF) (m/z) calcd for [C_8_H_4_NBr_3_O_2_ + Na]^+^: 405.76899; found: 405.78860.

### 4.3. Biological assay (Antioxidant activity)

Fe^3+^-reducing effects of the compounds were realized by Fe^3+^(CN-)_6_ reducing [46] as given previously [56]. Cu^2+^ reducing effect of the bromophenol compounds was realized according to a prior study [57] as detailed given [58,59]. FRAP method of the bromophenol compounds is realized by reducing of Fe^3+^-TPTZ complex in an acidic environment [60]. The DPPH radical removing effects of the bromophenol compounds were done according to the method of Blois [61] as given previously [62–64]. ABTS radical scavenging of bromophenol compounds is performed according to Gulcin’s methods [65–67]. The radical scavenging capacities (RSC) of the bromophenol compounds were calculated as follows: RSC (%) = (1-Ac/Ad) × 100 [4,68,69]. Where Ac and Ad are the absorbances of control and the compounds. IC_50_ was obtained from the graph, which plotted inhibition percentage against the bromophenol compounds concentrations (μg/mL) [35,70,71].

## Supplementary material contains NMR and HRMS spectra of synthesized compounds

^1^H-NMR spectrum of the compound **17** (400 MHz, CDCl_3_)

^13^C-NMR spectrum of the compound **17** (100 MHz, CDCl_3_).

HRMS spectrum of the compound **17.**

^1^H-NMR spectrum of the compound **18** (400 MHz, CDCl_3_).

^13^C-NMR spectrum of the compound **18** (100 MHz, CDCl_3_).

HRMS spectrum of the compound **18.**

^1^H-NMR spectrum of the compound **19** (400 MHz, CDCl_3_).

^13^C-NMR spectrum of the compound **19** (100 MHz, CDCl_3_).

HRMS spectrum of the compound **19.**

^1^H-NMR spectrum of the compound **20** (400 MHz, CDCl_3_).

^13^C-NMR spectrum of the compound **20** (100 MHz, CDCl_3_).

HRMS spectrum of the compound **20.**

^1^H-NMR spectrum of the compound **21** (400 MHz, CDCl_3_).

^13^C-NMR spectrum of the compound **21** (100 MHz, CDCl_3_).

HRMS spectrum of the compound **21.**

^1^H-NMR spectrum of the compound **22** (400 MHz, CDCl_3_).

^13^C-NMR spectrum of the compound **22** (100 MHz, CDCl_3_).

HRMS spectrum of the compound **22.**

^1^H-NMR spectrum of the compound **23** (400 MHz, acetone-d_6_).

^13^C-NMR spectrum of the compound **23** (100 MHz, acetone-d_6_).

HRMS spectrum of the compound **23.**

^1^H-NMR spectrum of the compound **24** (400 MHz, CDCl_3_).

^13^C-NMR spectrum of the compound **24** (100 MHz, CDCl_3_).

HRMS spectrum of the compound **24.**

^13^C-NMR spectrum of the compound **25** (100 MHz, acetone-d_6_).

^1^H-NMR spectrum of the compound **25** (400 MHz, acetone-d_6_).

HRMS spectrum of the compound **25.**

^1^H-NMR spectrum of the natural product **1** (400 MHz, acetone-d_6_).

^13^C-NMR spectrum of the natural product **1** (100 MHz, acetone-d_6_).

HRMS spectrum of the compound **1.**

^1^H-NMR spectrum of the natural product **2** (400 MHz, acetone-d_6_).

^13^C-NMR spectrum of the natural product **2** (100 MHz, acetone-d_6_).

HRMS spectrum of the compound **2.**

^1^H-NMR spectrum of the compound **26** (400 MHz, acetone-d_6_).

^13^C-NMR spectrum of the compound **26** (100 MHz, acetone-d_6_).

HRMS spectrum of the compound **26.**

^1^H-NMR spectrum of the compound **27** (400 MHz, acetone-d_6_).

^13^C-NMR spectrum of the compound **27** (100 MHz, acetone-d_6_).

HRMS spectrum of the compound **27.**

^1^H-NMR spectrum of the compound **28** (400 MHz, acetone-d_6_).

^13^C-NMR spectrum of the compound **28** (100 MHz, acetone-d_6_).

HRMS spectrum of the compound **28.**

## Figures and Tables

**Table 1:** Fe^3+^, Cu^2+^ and Fe^3+^-TPTZ reducing ability of bromophenol derivatives **1, 2**, and **17–28** and standards at 60 μg/mL concentration

Antioxidants	Fe^3+^ reducing		Cu^2+^ reducing		Fe^3+^-TPTZ reducing	
	λ_700_	r^2^	λ _450_	r^2^	λ _593_	r^2^
**BHA**	1.744 ± 0.004	0.7114	1.864 ± 0.015	0.9854	2.254 ± 0.004	0.7435
**BHT**	1.563 ± 0.003	0.8358	1.774 ± 0.003	0.7642	2.146 ± 0.002	0.8599
α-Tocopherol	1.473 ± 0.003	0.9499	1.795 ± 0.003	0.9747	1.497 ± 0.002	0.8531
**Trolox**	1.648 ± 0.007	0.8992	1.829 ± 0.004	0.6061	1.993 ± 0.004	0.9494
**1**	1.775 ± 0.003	0.6791	1.896 ± 0.002	0.6828	1.996 ± 0.002	0.7367
**2**	0.877 ± 0.020	0.9463	1.345 ± 0.005	0.9507	0.744 ± 0.002	0.8929
**17**	0.312 ± 0.009	0.9023	0.303 ± 0.002	0.7484	0.453 ± 0.002	0.8902
**18**	0.445 ± 0.001	0.9557	0.506 ± 0.010	0.5767	0.404 ± 0,004	0.9627
**19**	0.377 ± 0.003	0.9544	0.430 ± 0.009	0.8159	0.407 ± 0.002	0.7252
**20**	0.355 ± 0.001	0.9542	0.716 ± 0.002	0.7199	0.344 ± 0.002	0.9844
**21**	0.355 ± 0.003	0.9290	0.666 ± 0.002	0.8792	0.667 ± 0.002	0.8175
**22**	0.253 ± 0.004	0.9321	0.265 ± 0.001	0.9579	0.304 ± 0.004	0.9683
**23**	0.302 ± 0.001	0.9460	0.726 ± 0.002	0.8705	0.403 ± 0.003	0.9321
**24**	0.463 ± 0.010	0.9264	0.665 ± 0.003	0.9843	0.578 ± 0.010	0.8693
**25**	1.743 ± 0.002	0.6555	1.853 ± 0.003	0.6573	2.455 ± 0.004	0.8362
**26**	1.704 ± 0.002	0.7336	1.642 ± 0.002	0,9589	2.016 ± 0.002	0.7929
**27**	1.663 ± 0.003	0.8012	1.538 ± 0.110	0.9015	1.894 ± 0.002	0.8755
**28**	1.856 ± 0.002	0.8206	1.609 ± 0.120	0.9589	1.605 ± 0.001	0.8889

**Table 2:** Half maximal scavenging concentration (IC_50_, μg/mL) for DPPH^•^ scavenging and ABTS^•+^ scavenging effects of bromophenol derivatives **1, 2**, and **17–28** and standards.

Compounds	DPPH• scavenging		ABTS^•+^ scavenging	
	IC_50_^*^	*r* ^2^	IC_50_^*^	*r* ^2^
**BHA**	14.74	0.7129	11.17	0.9030
**BHT**	15.75	0.9986	4.12	0.9690
α-Tocopherol	12.15	0.7950	23.89	0.9732
**Trolox**	9.36	0.6575	11.75	0.8513
**1**	9.90	0.6119	6.41	0.9961
**2**	10.66	0.8511	30.13	0.9457
**17**	173.25	0.9145	173.25	0.8727
**18**	231.00	0.7611	346.50	0.9371
**19**	17.32	0.9865	231.00	0.9134
**20**	346.50	0.9735	115.50	0.8320
**21**	231.00	0.8642	173.25	0.9078
**22**	138.60	0.9851	173.25	0.9722
**23**	33.00	0.8860	231.00	0.9675
**24**	138.60	0.9810	138.60	0.8109
**25**	9.36	0.6059	4.27	0.9016
**26**	9.49	0.8680	231.00	0.9652
**27**	10.28	0.6654	6.86	0.9018
**28**	10.19	0.6496	10.66	0.9652
