# Supplementary figures and images for: Synthesis and antioxidant activities of benzylic bromophenols inclusive of natural products
Source: Turk J Chem. 2022 May 6;46(5):1405–16. doi: 10.55730/1300-0527.3447 (PMC10390195; doi:10.55730/1300-0527.3447)

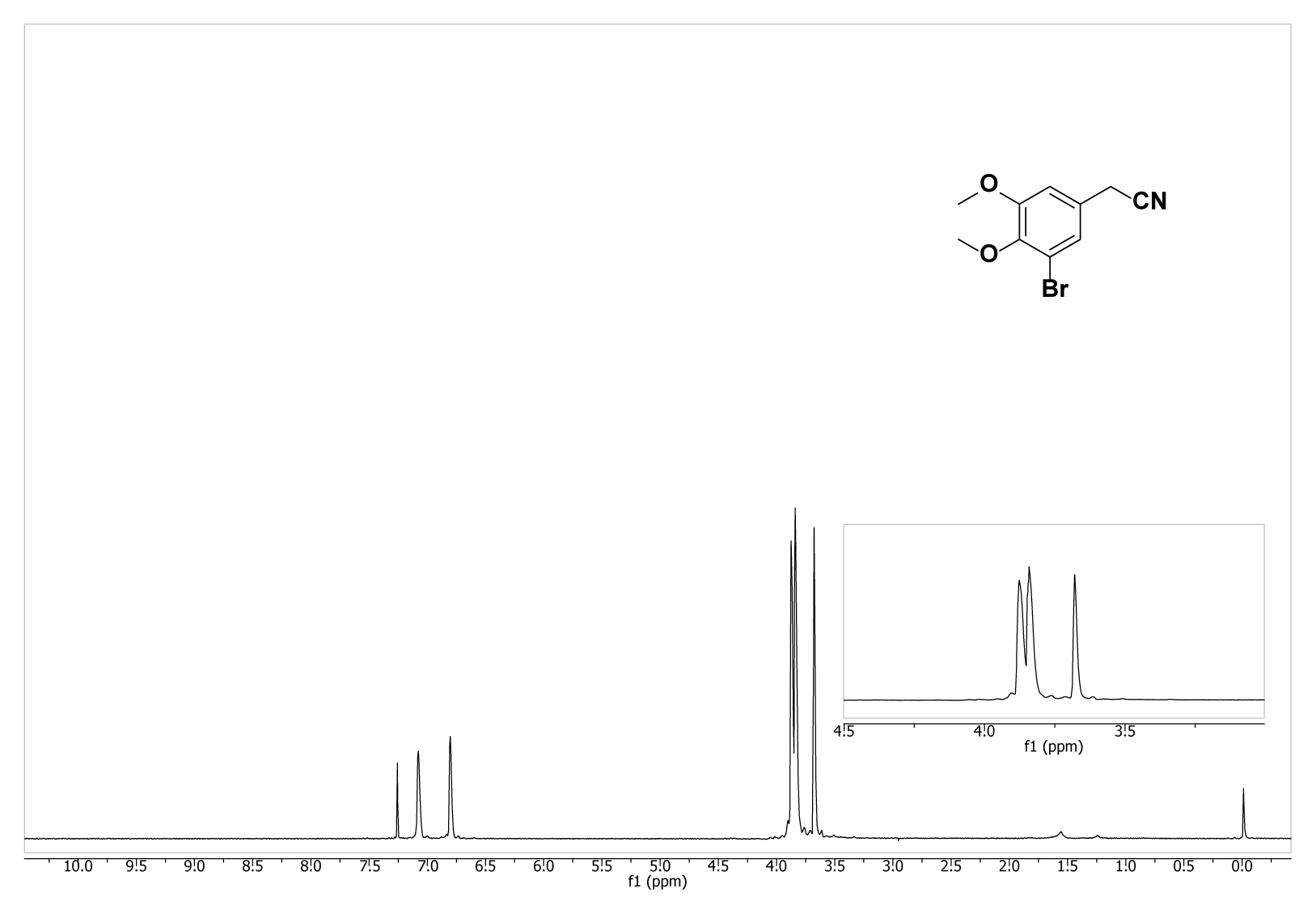

Supplement: Supplementary file 1 — 1H-NMR spectrum of the compound 17 (400 MHz, CDCl3) [file turkjchem-46-5-1405s1.tif]

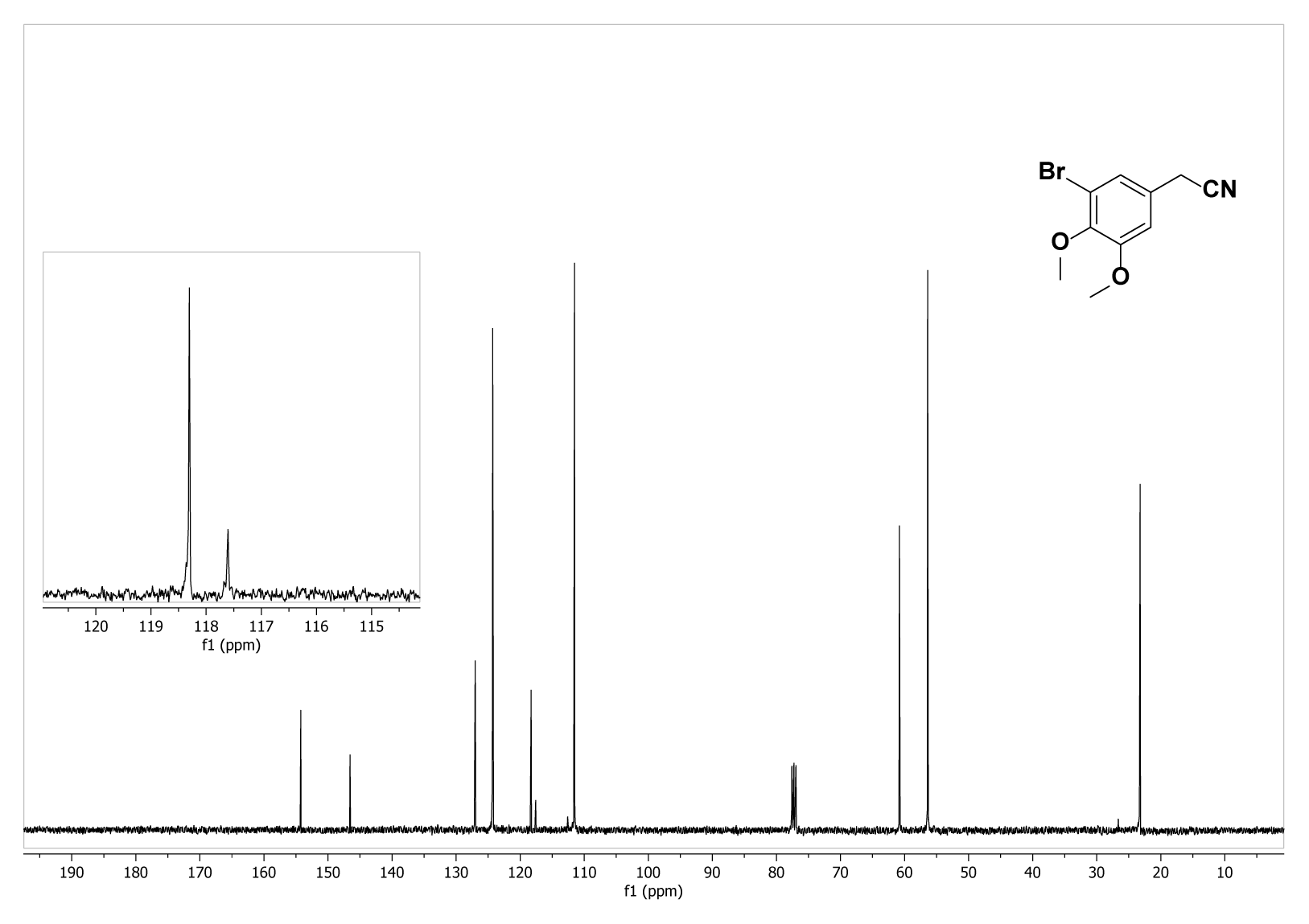

Supplement: Supplementary file 2 — 13C-NMR spectrum of the compound 17 (100 MHz, CDCl3). [file turkjchem-46-5-1405s2.tif]

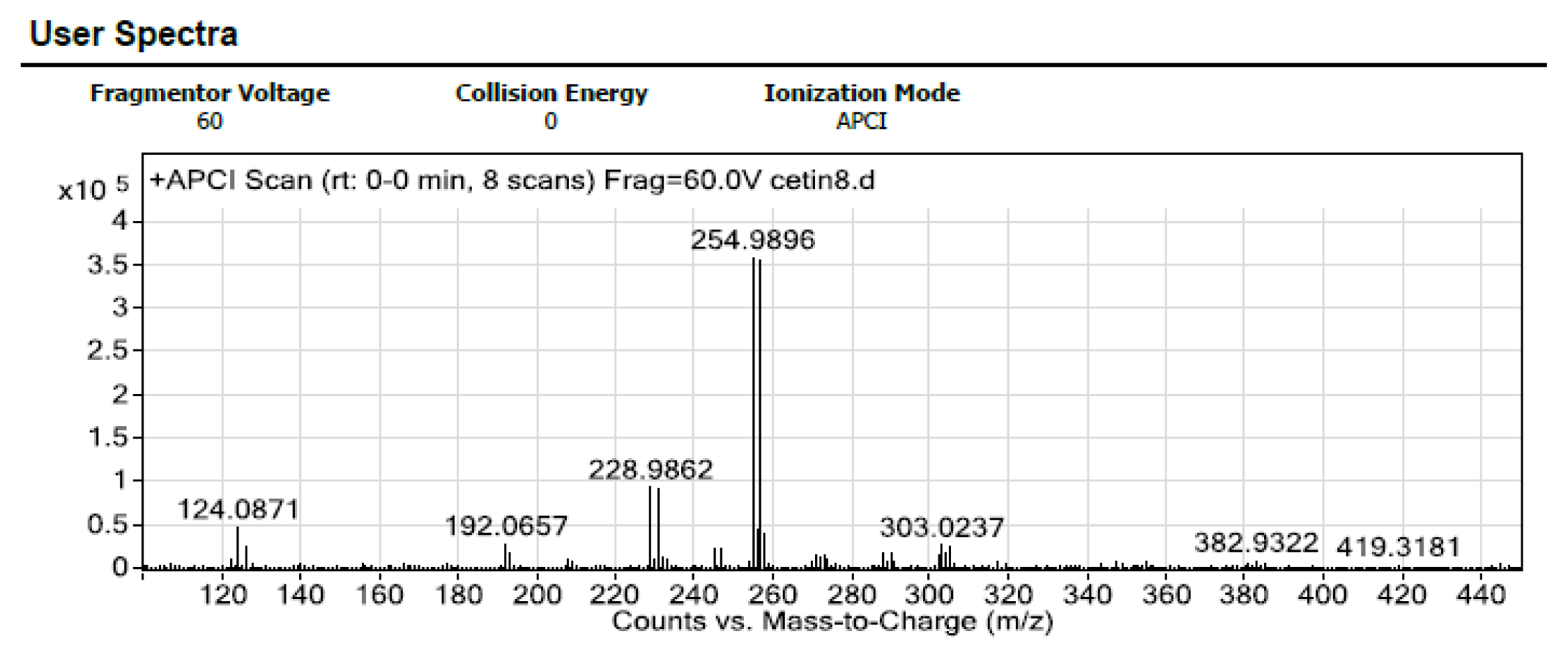

Supplement: Supplementary file 3 — HRMS spectrum of the compound 17. [file turkjchem-46-5-1405s3.tif]

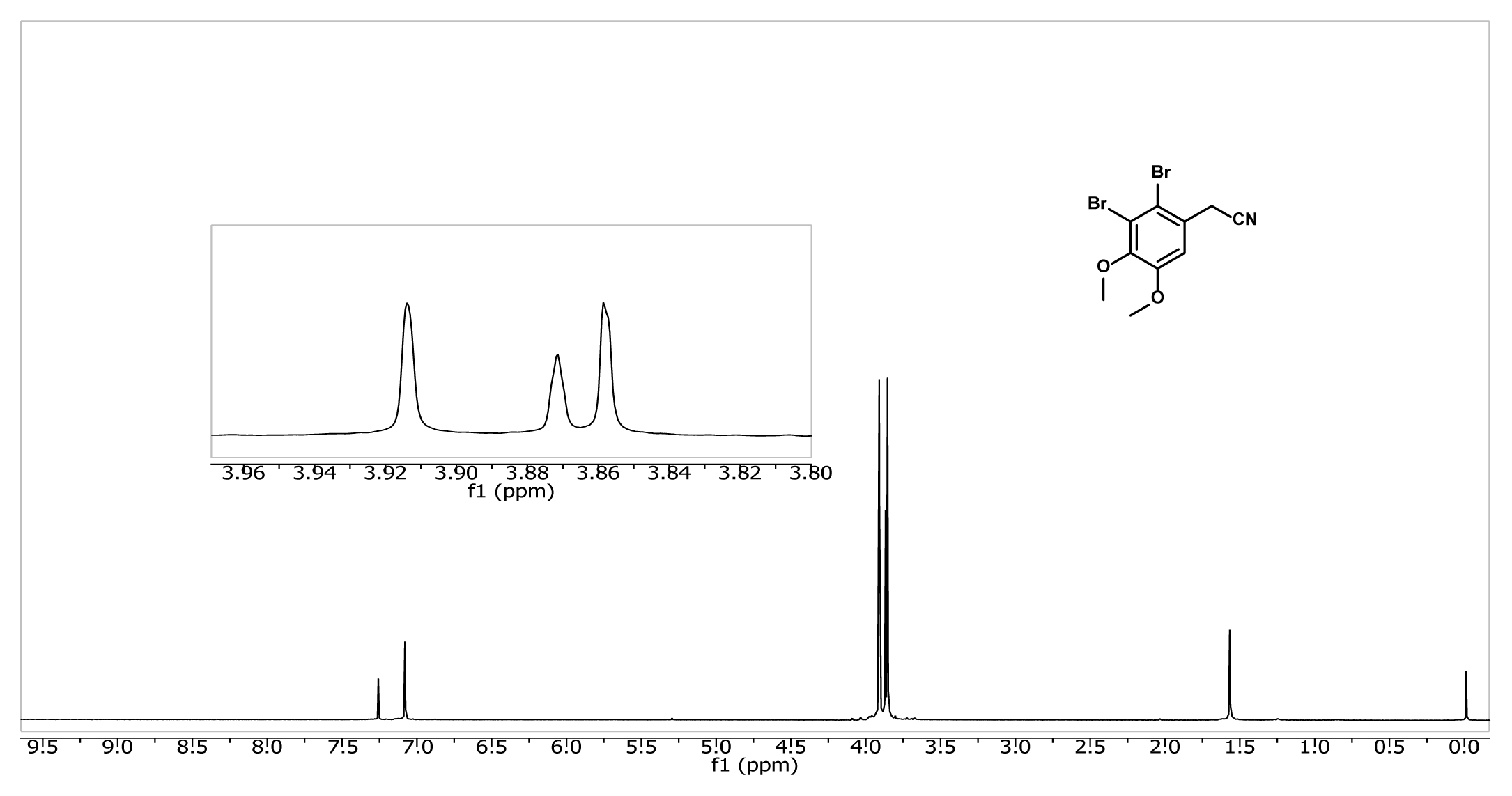

Supplement: Supplementary file 4 — 1H-NMR spectrum of the compound 18 (400 MHz, CDCl3). [file turkjchem-46-5-1405s4.tif]

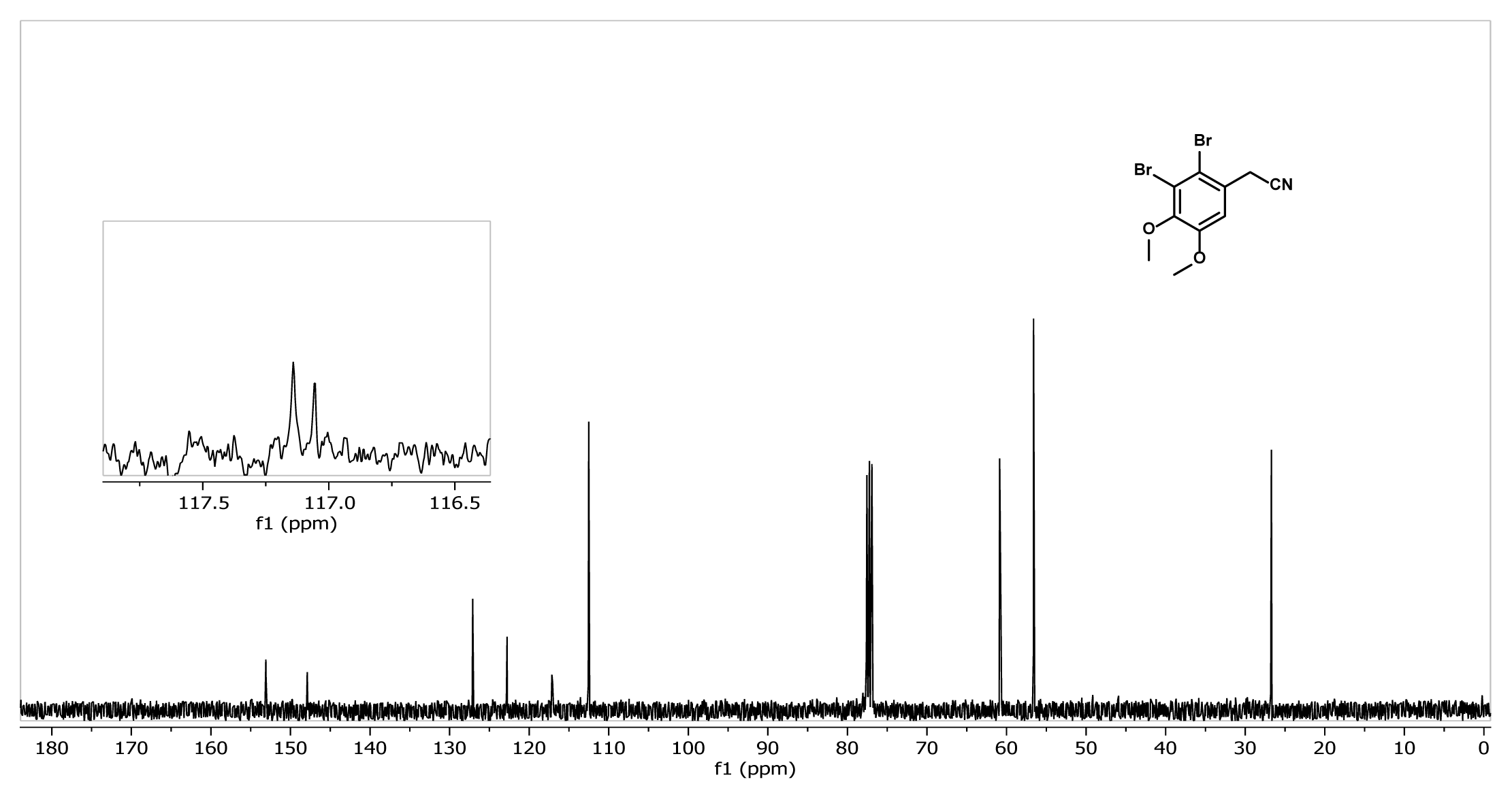

Supplement: Supplementary file 5 — 13C-NMR spectrum of the compound 18 (100 MHz, CDCl3). [file turkjchem-46-5-1405s5.tif]

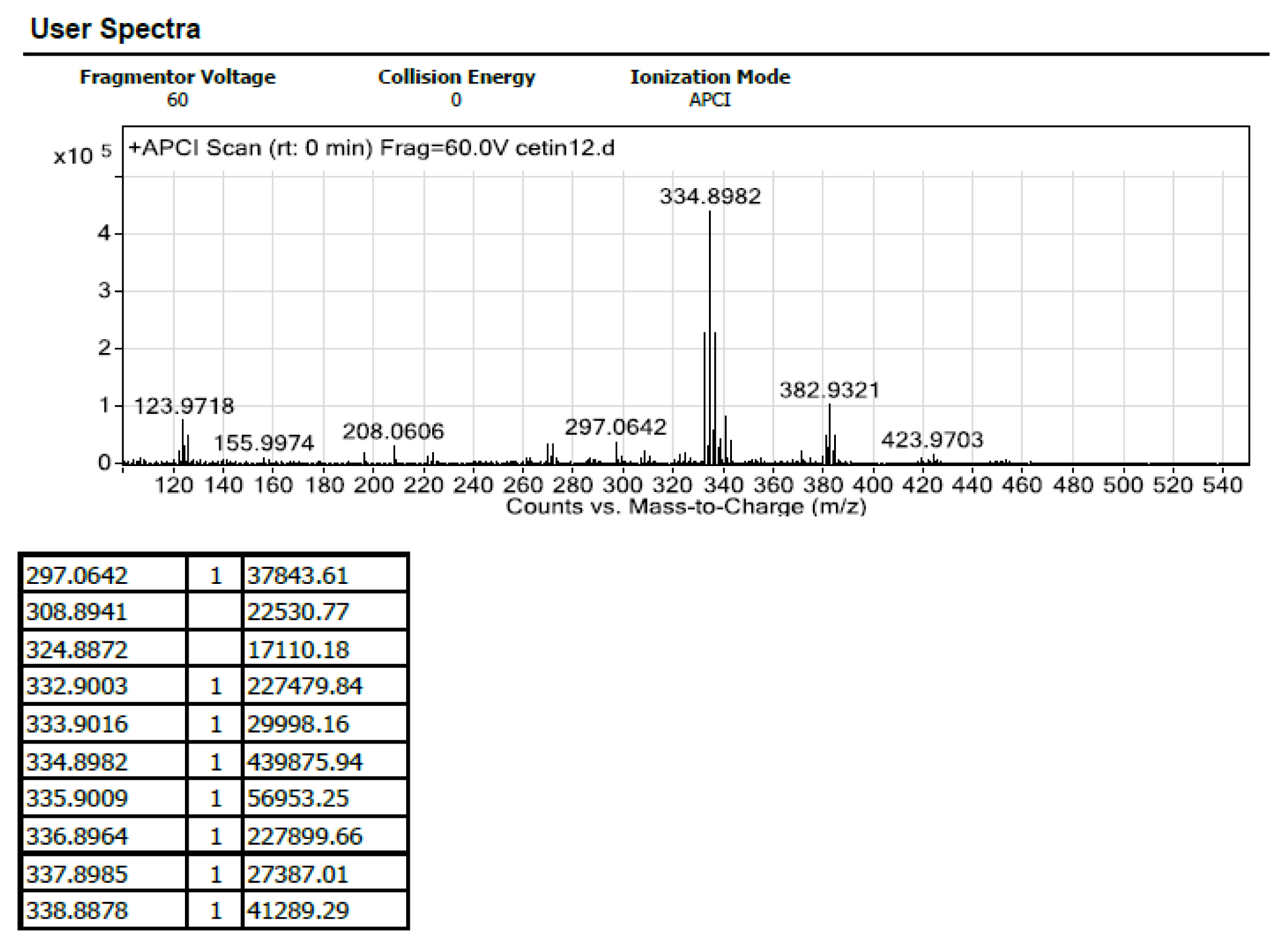

Supplement: Supplementary file 6 — HRMS spectrum of the compound 18. [file turkjchem-46-5-1405s6.tif]

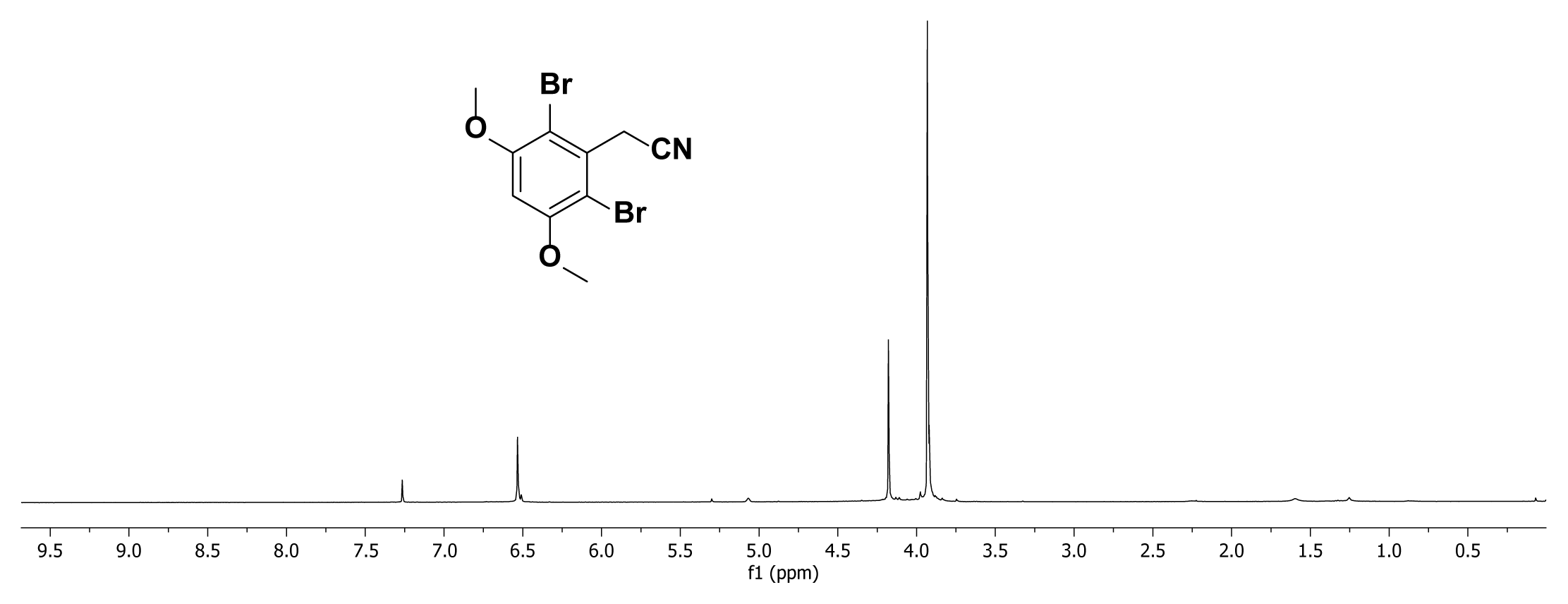

Supplement: Supplementary file 7 — 1H-NMR spectrum of the compound 19 (400 MHz, CDCl3). [file turkjchem-46-5-1405s7.tif]

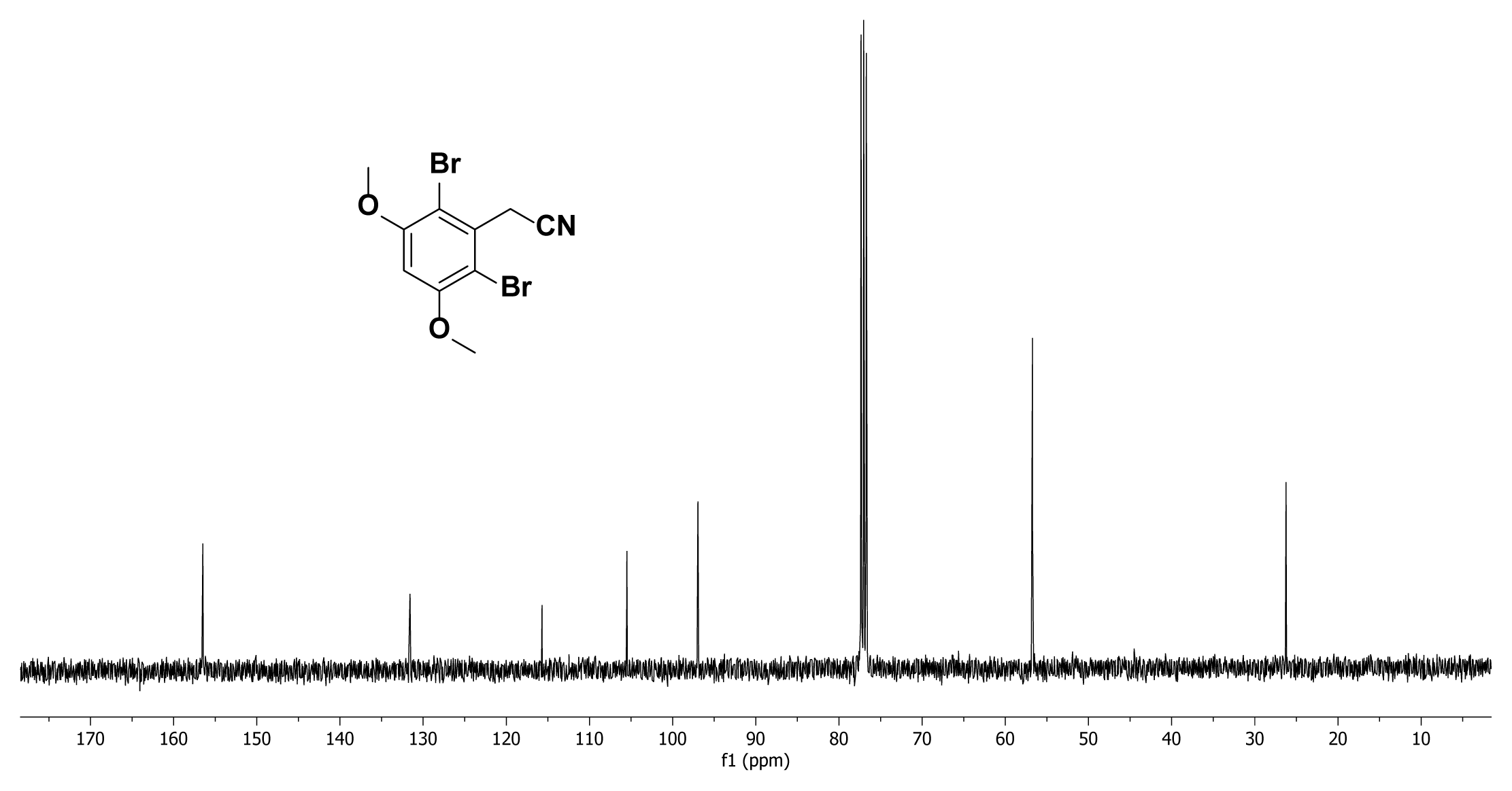

Supplement: Supplementary file 8 — 13C-NMR spectrum of the compound 19 (100 MHz, CDCl3). [file turkjchem-46-5-1405s8.tif]

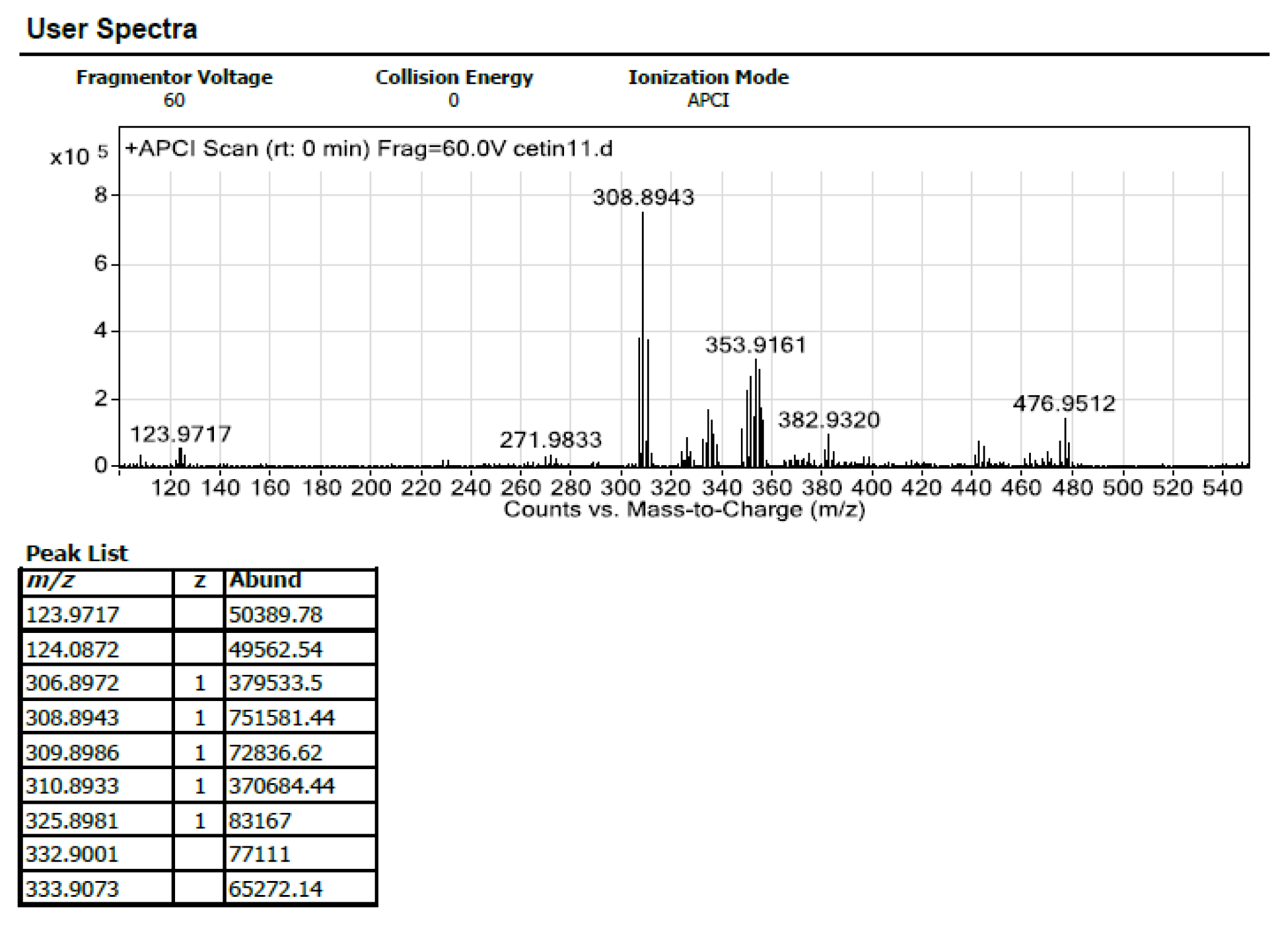

Supplement: Supplementary file 9 — HRMS spectrum of the compound 19. [file turkjchem-46-5-1405s9.tif]

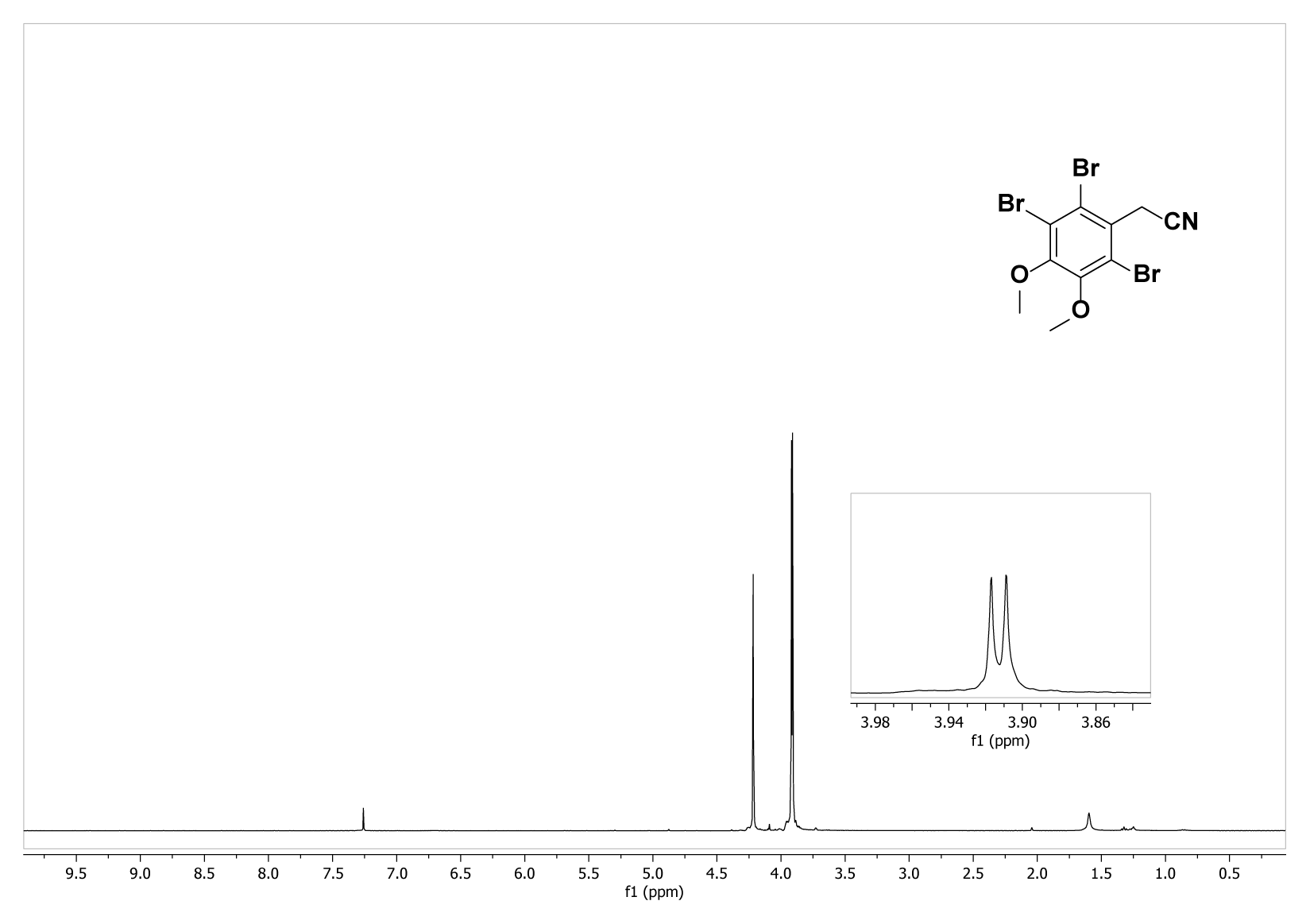

Supplement: Supplementary file 10 — 1H-NMR spectrum of the compound 20 (400 MHz, CDCl3). [file turkjchem-46-5-1405s10.tif]

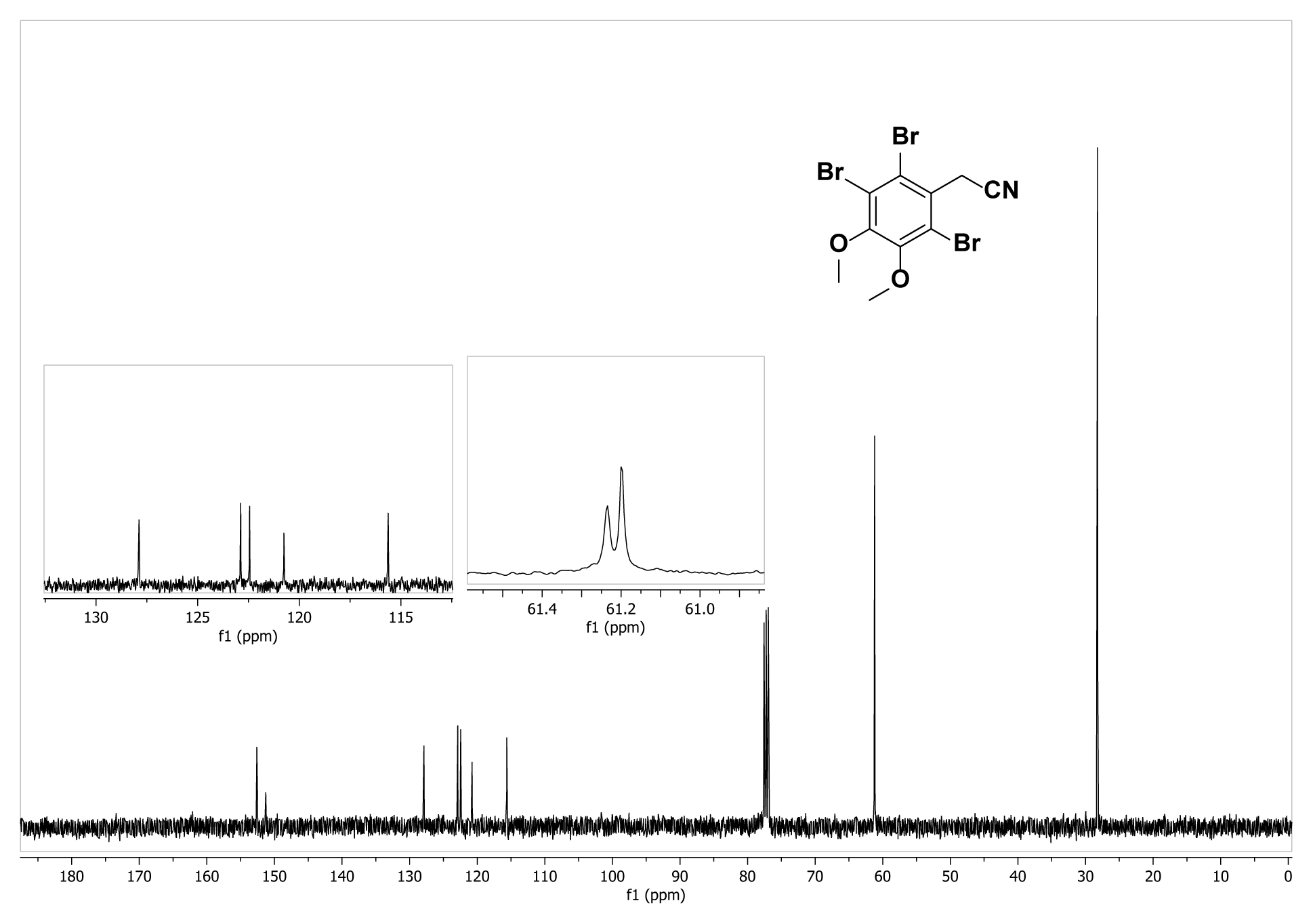

Supplement: Supplementary file 11 — 13C-NMR spectrum of the compound 20 (100 MHz, CDCl3). [file turkjchem-46-5-1405s11.tif]

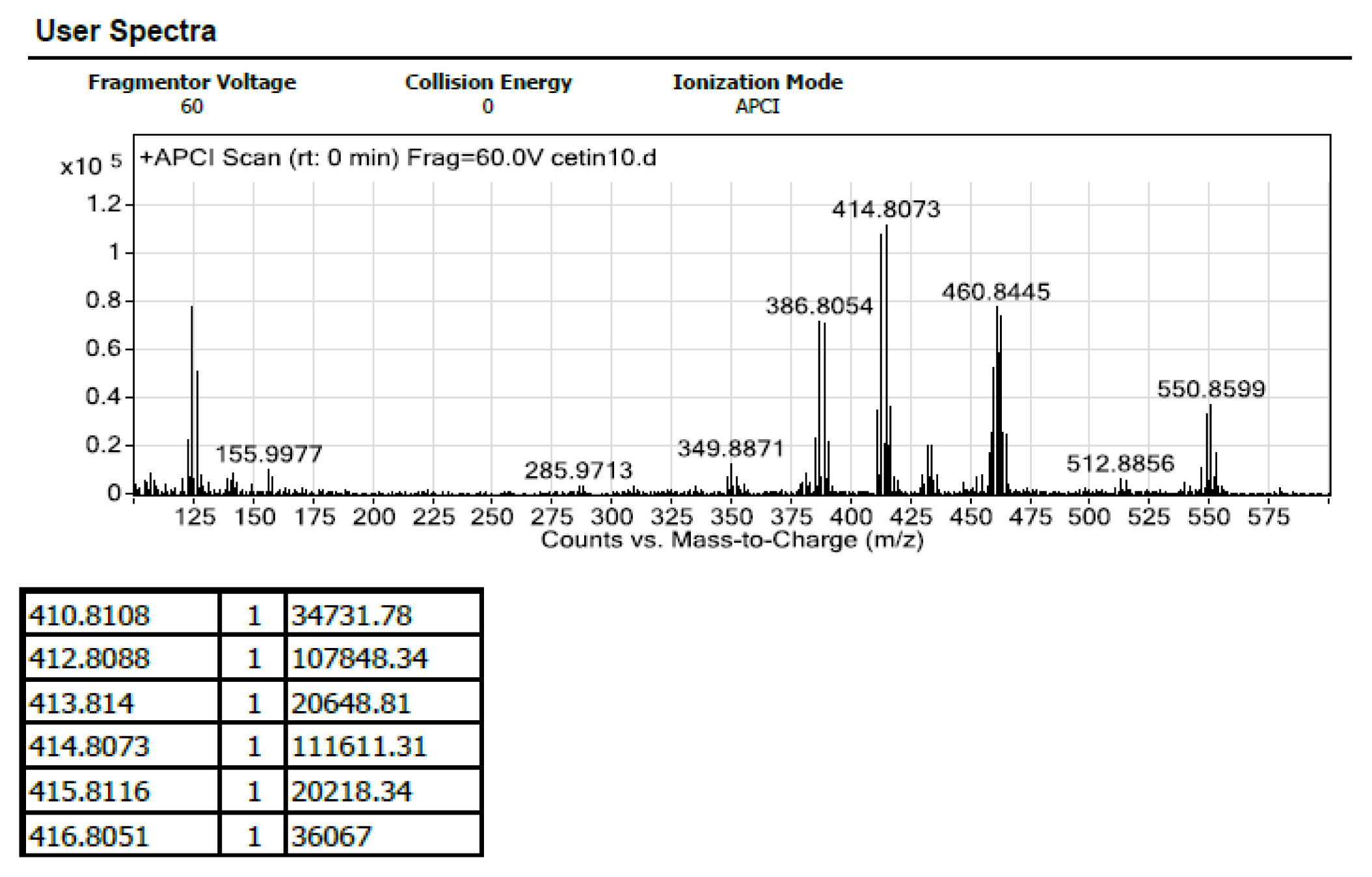

Supplement: Supplementary file 12 — HRMS spectrum of the compound 20. [file turkjchem-46-5-1405s12.tif]

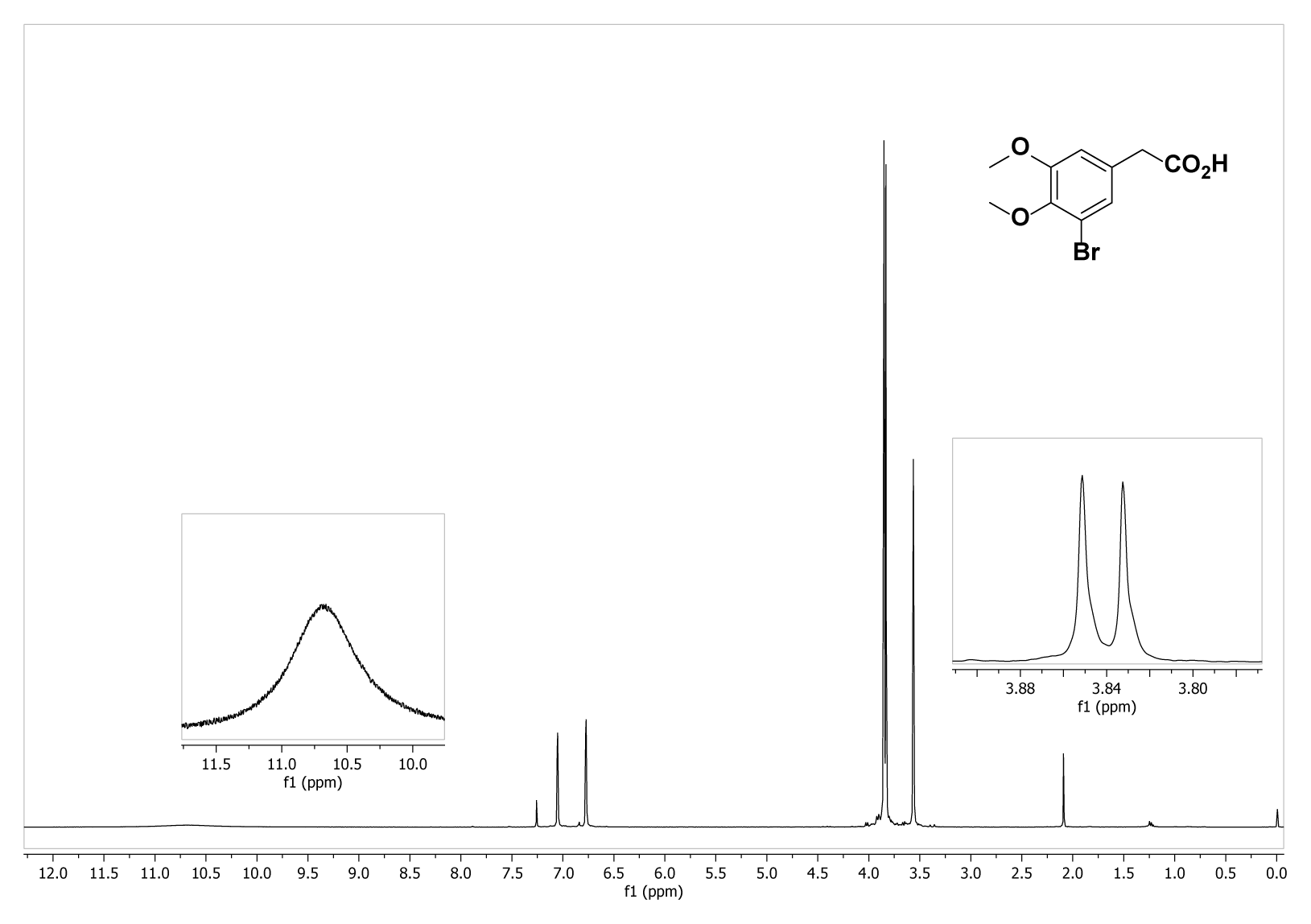

Supplement: Supplementary file 13 — 1H-NMR spectrum of the compound 21 (400 MHz, CDCl3). [file turkjchem-46-5-1405s13.tif]

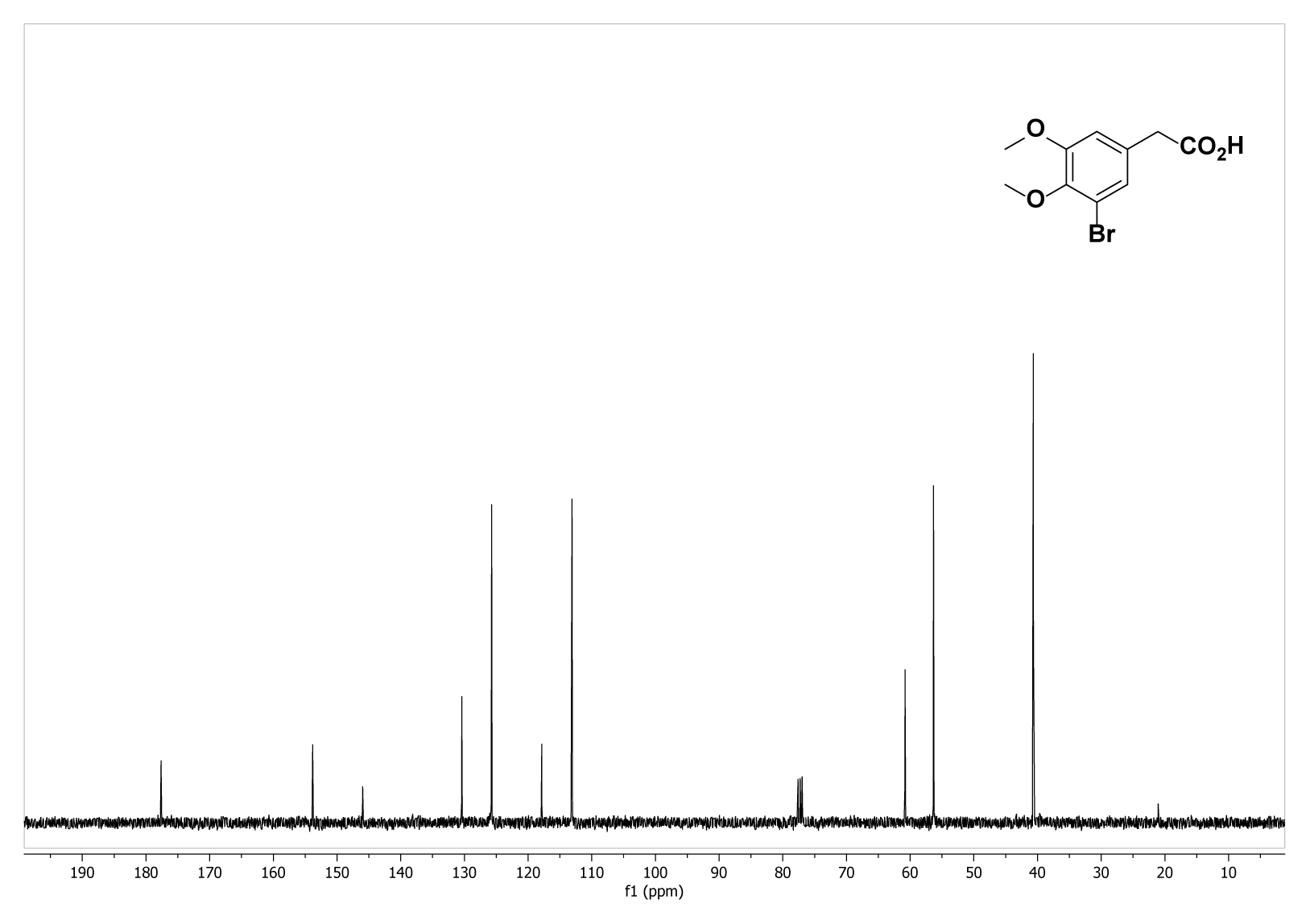

Supplement: Supplementary file 14 — 13C-NMR spectrum of the compound 21 (100 MHz, CDCl3). [file turkjchem-46-5-1405s14.tif]

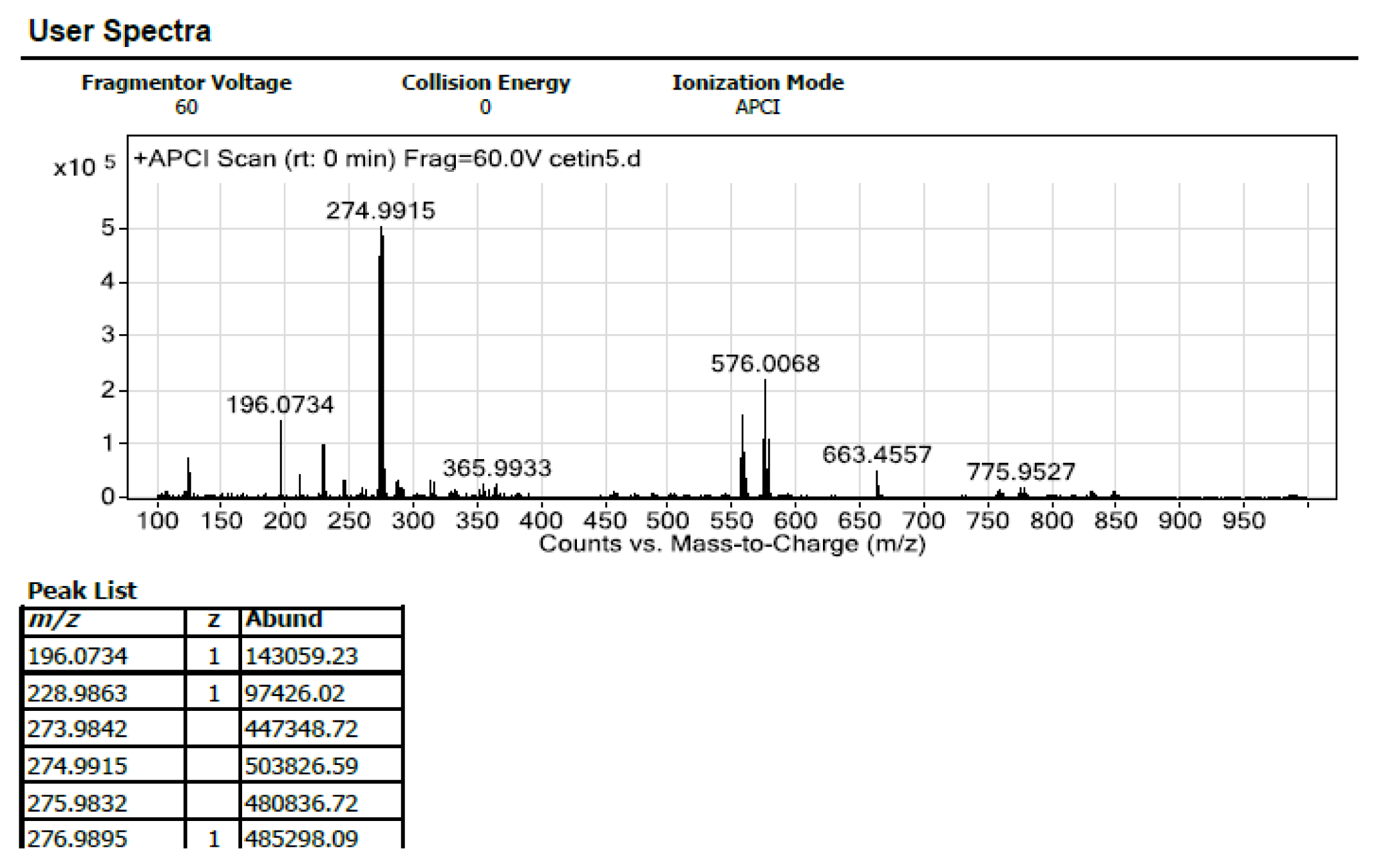

Supplement: Supplementary file 15 — HRMS spectrum of the compound 21. [file turkjchem-46-5-1405s15.tif]

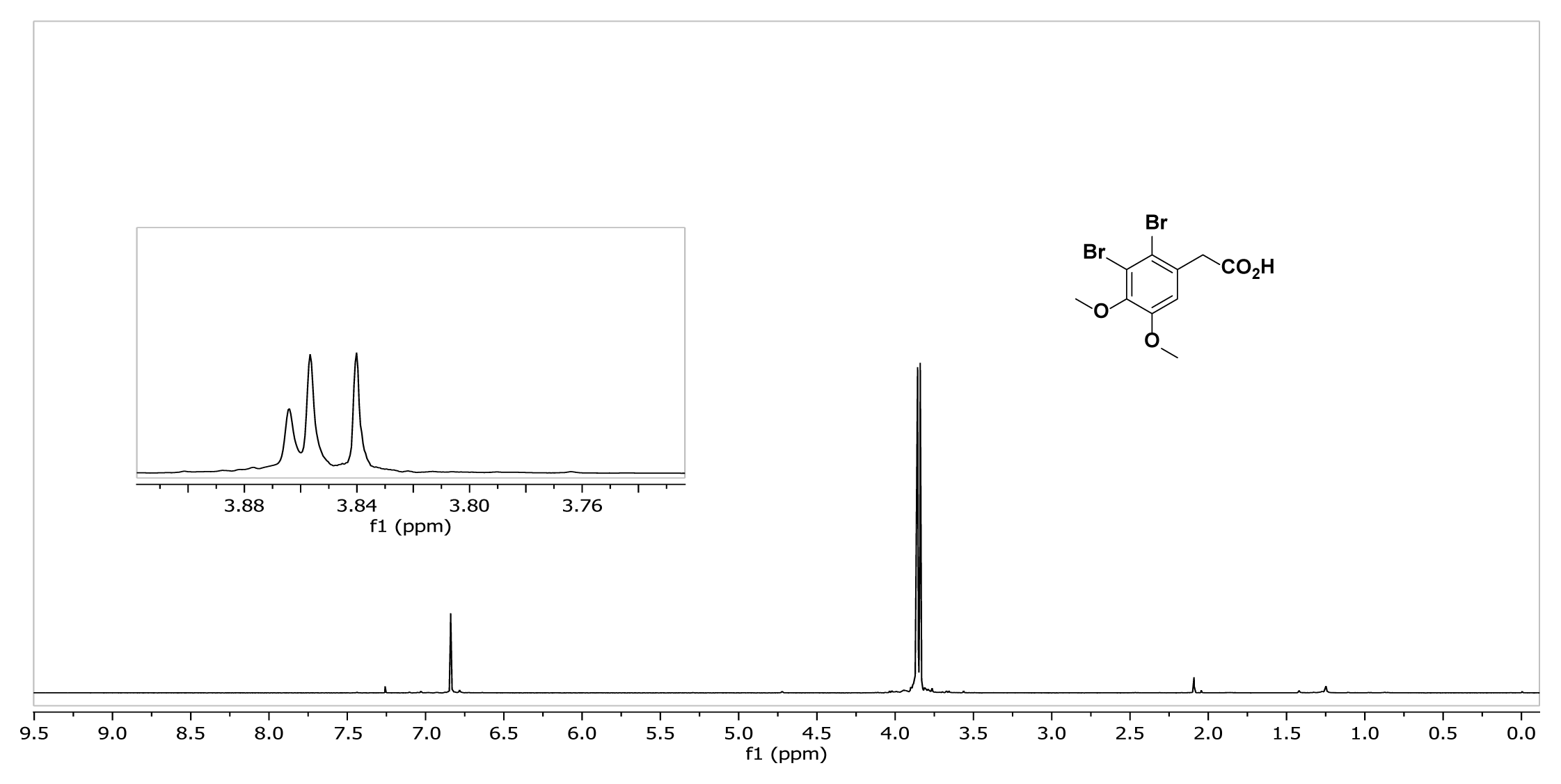

Supplement: Supplementary file 16 — 1H-NMR spectrum of the compound 22 (400 MHz, CDCl3). [file turkjchem-46-5-1405s16.tif]

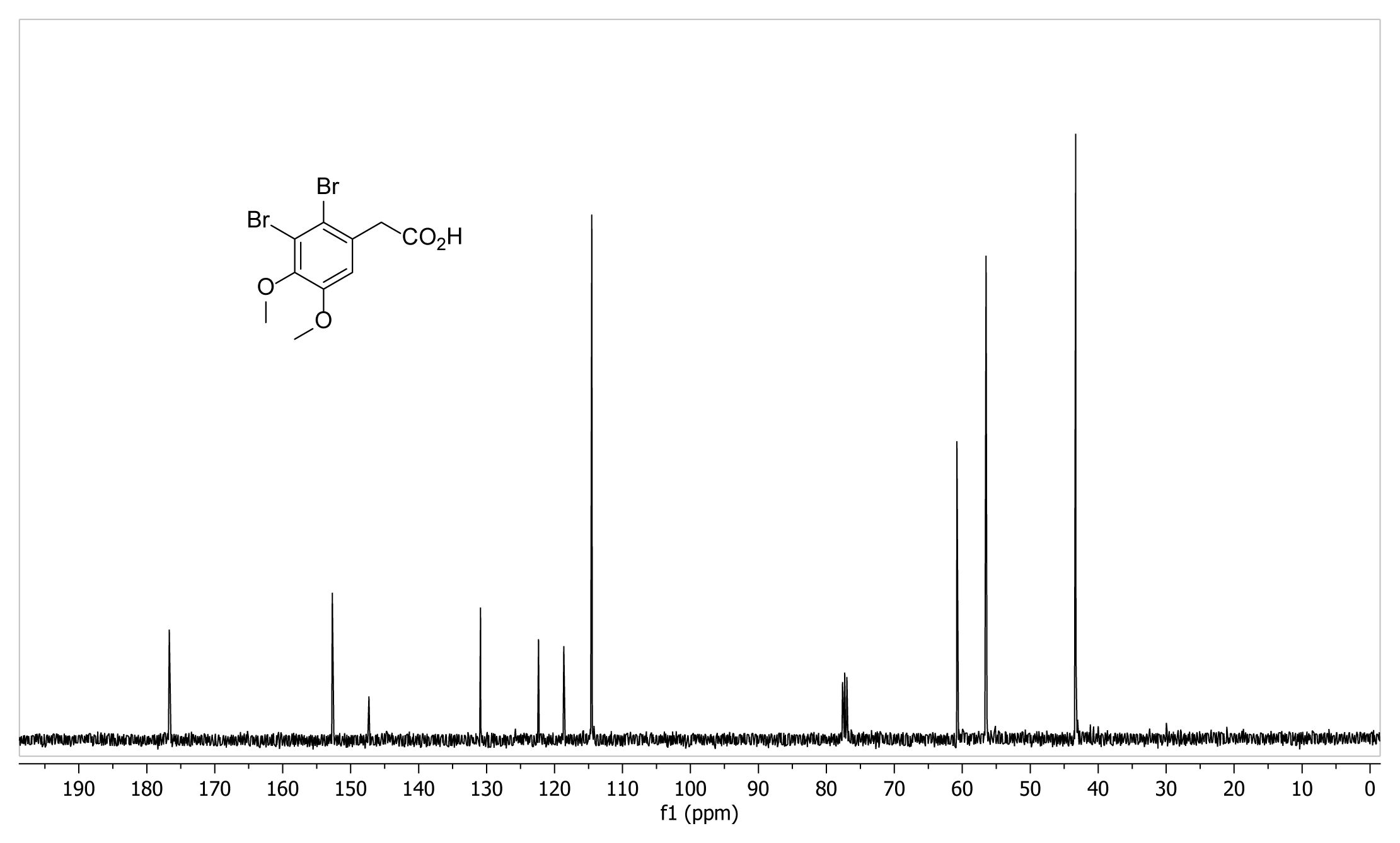

Supplement: Supplementary file 17 — 13C-NMR spectrum of the compound 22 (100 MHz, CDCl3). [file turkjchem-46-5-1405s17.tif]

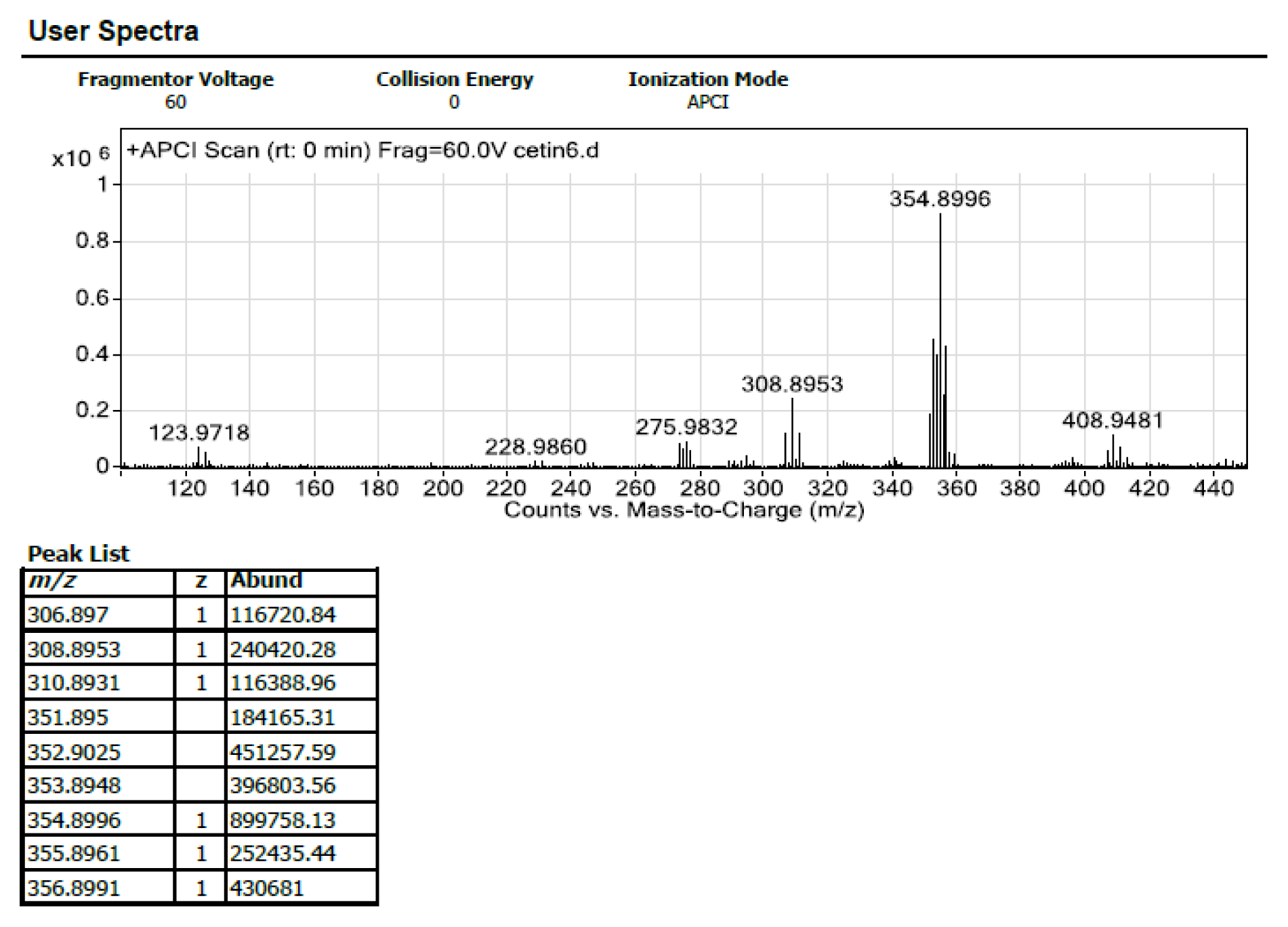

Supplement: Supplementary file 18 — HRMS spectrum of the compound 22. [file turkjchem-46-5-1405s18.tif]

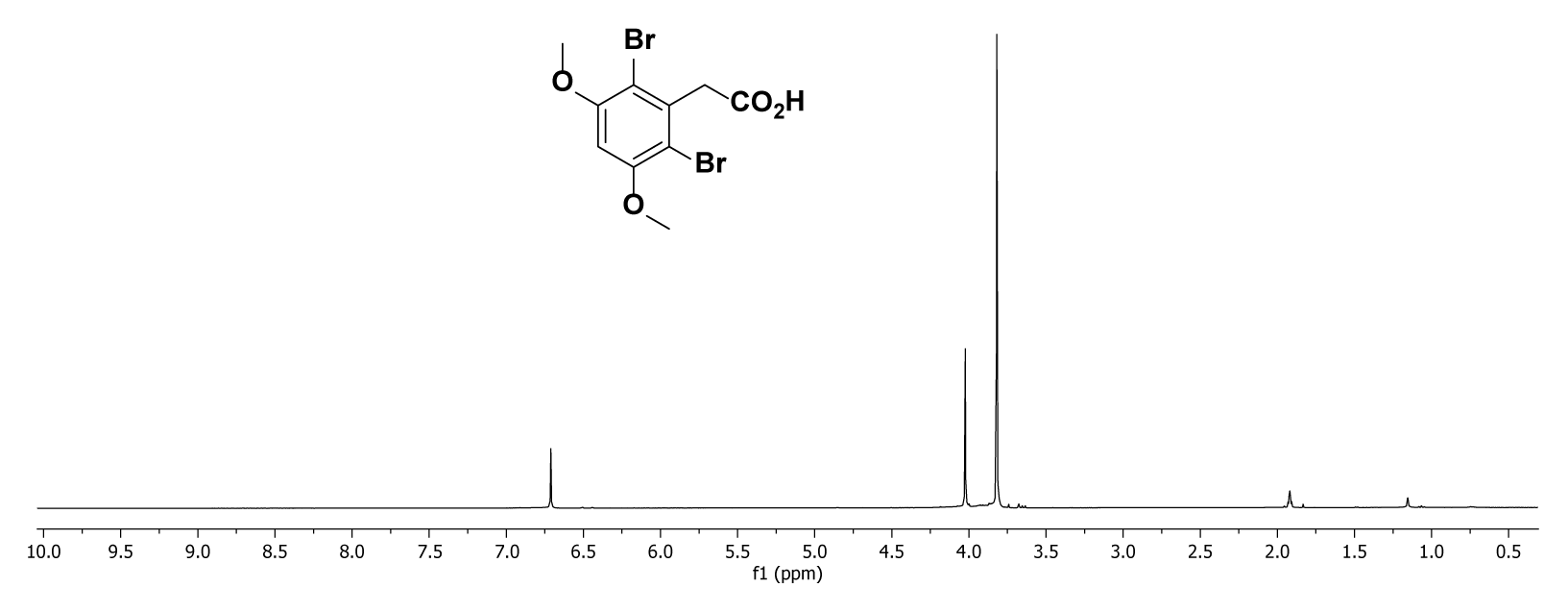

Supplement: Supplementary file 19 — 1H-NMR spectrum of the compound 23 (400 MHz, acetone-d6). [file turkjchem-46-5-1405s19.tif]

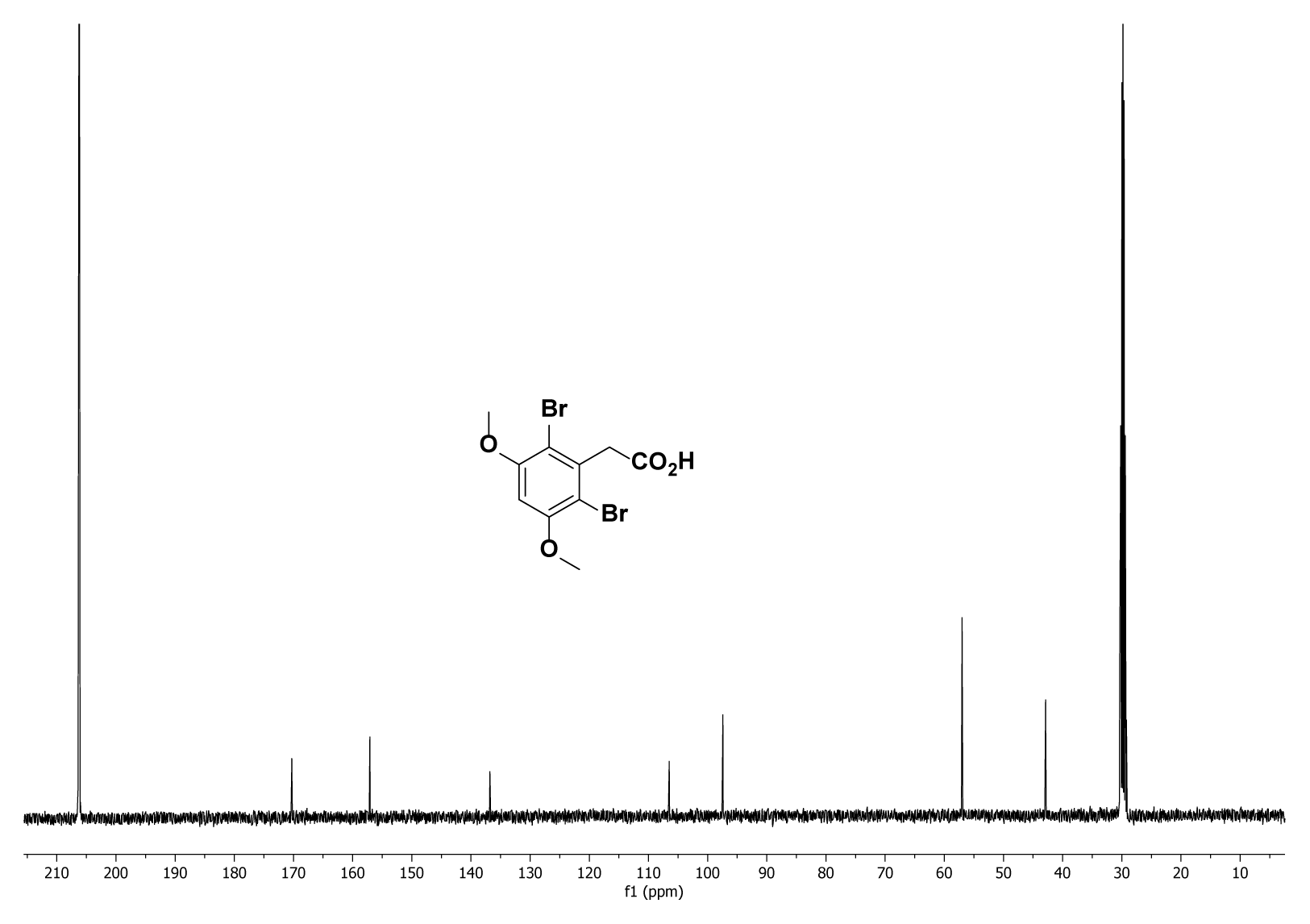

Supplement: Supplementary file 20 — 13C-NMR spectrum of the compound 23 (100 MHz, acetone-d6). [file turkjchem-46-5-1405s20.tif]

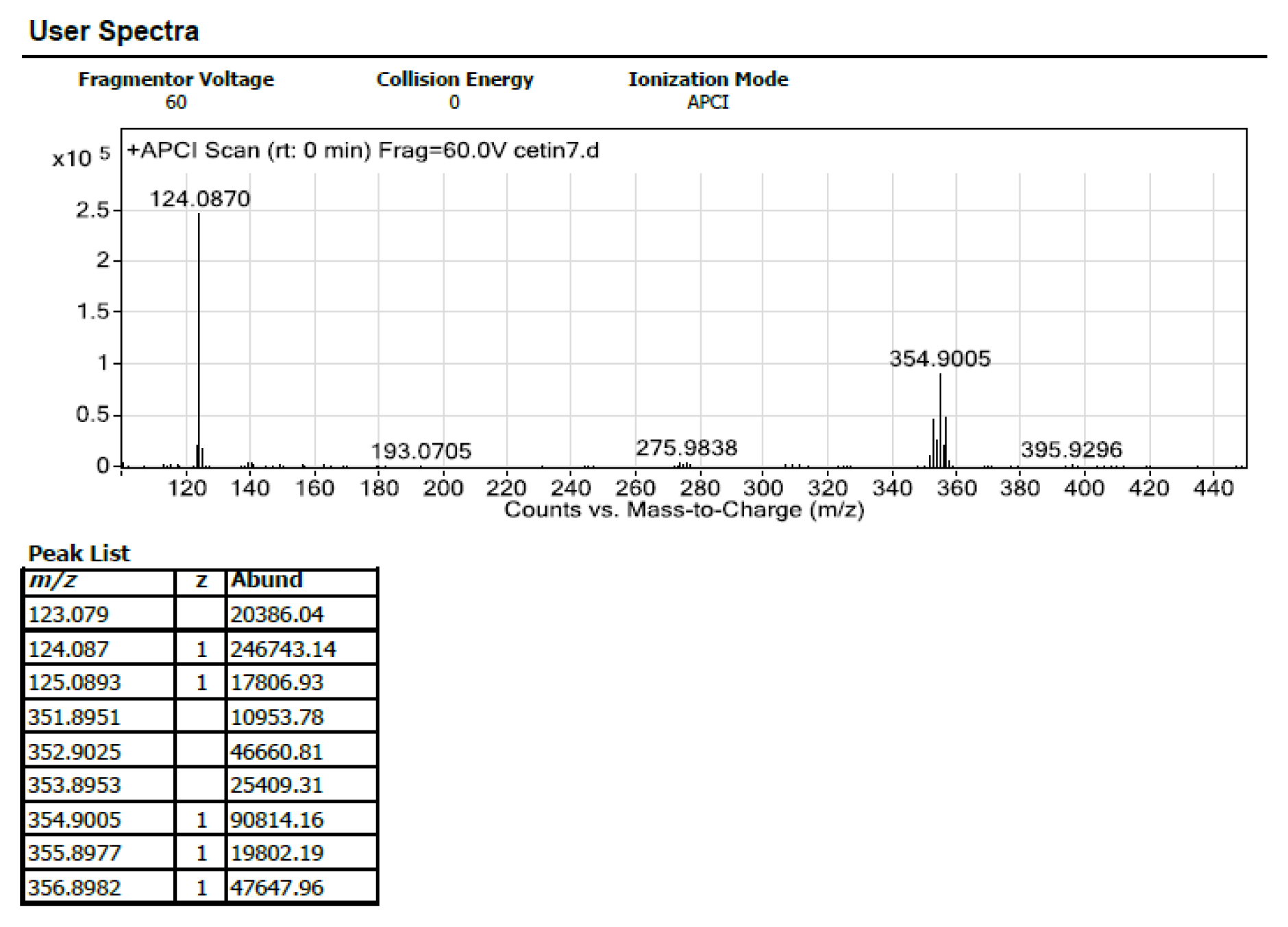

Supplement: Supplementary file 21 — HRMS spectrum of the compound 23. [file turkjchem-46-5-1405s21.tif]

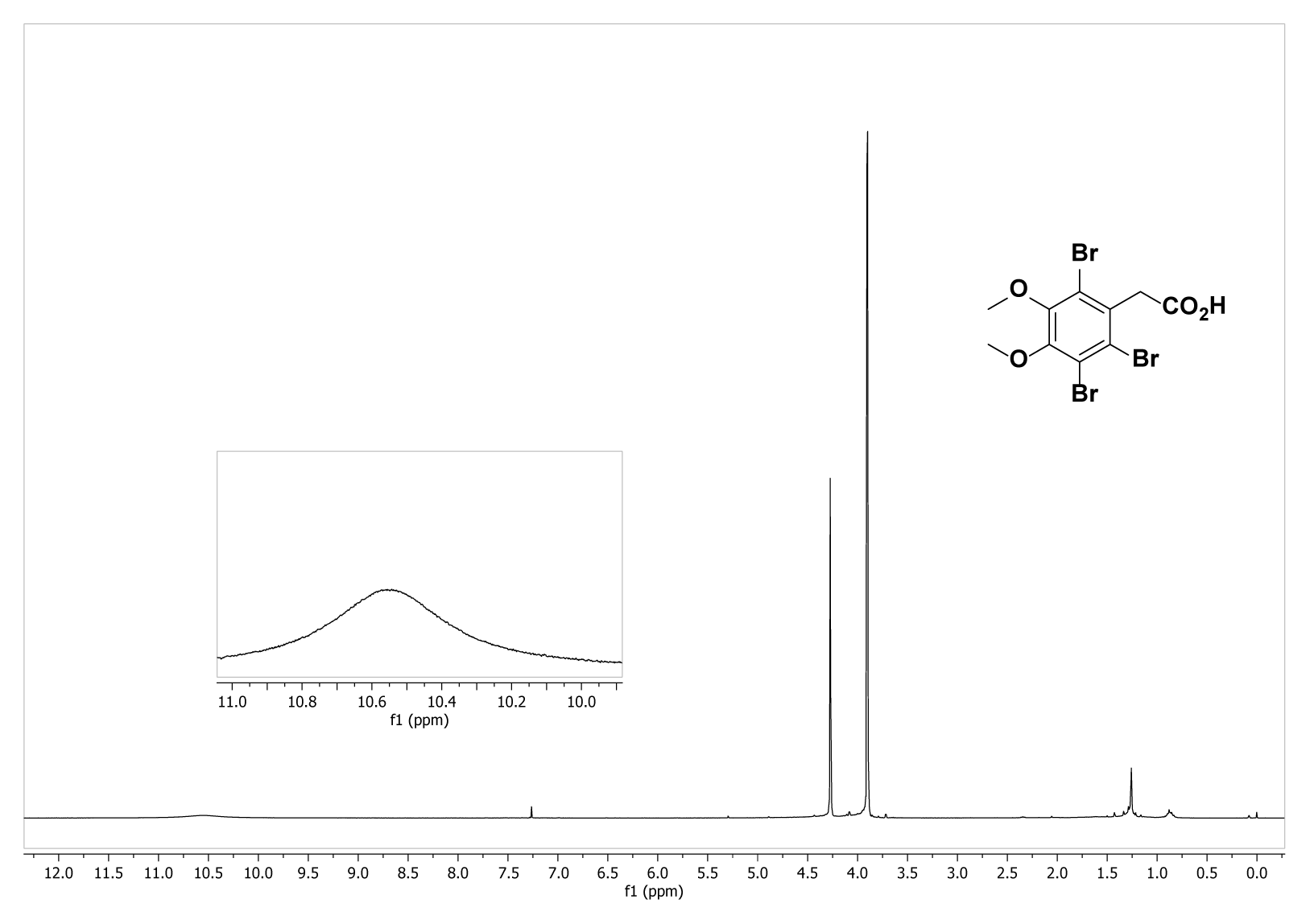

Supplement: Supplementary file 22 — 1H-NMR spectrum of the compound 24 (400 MHz, CDCl3). [file turkjchem-46-5-1405s22.tif]

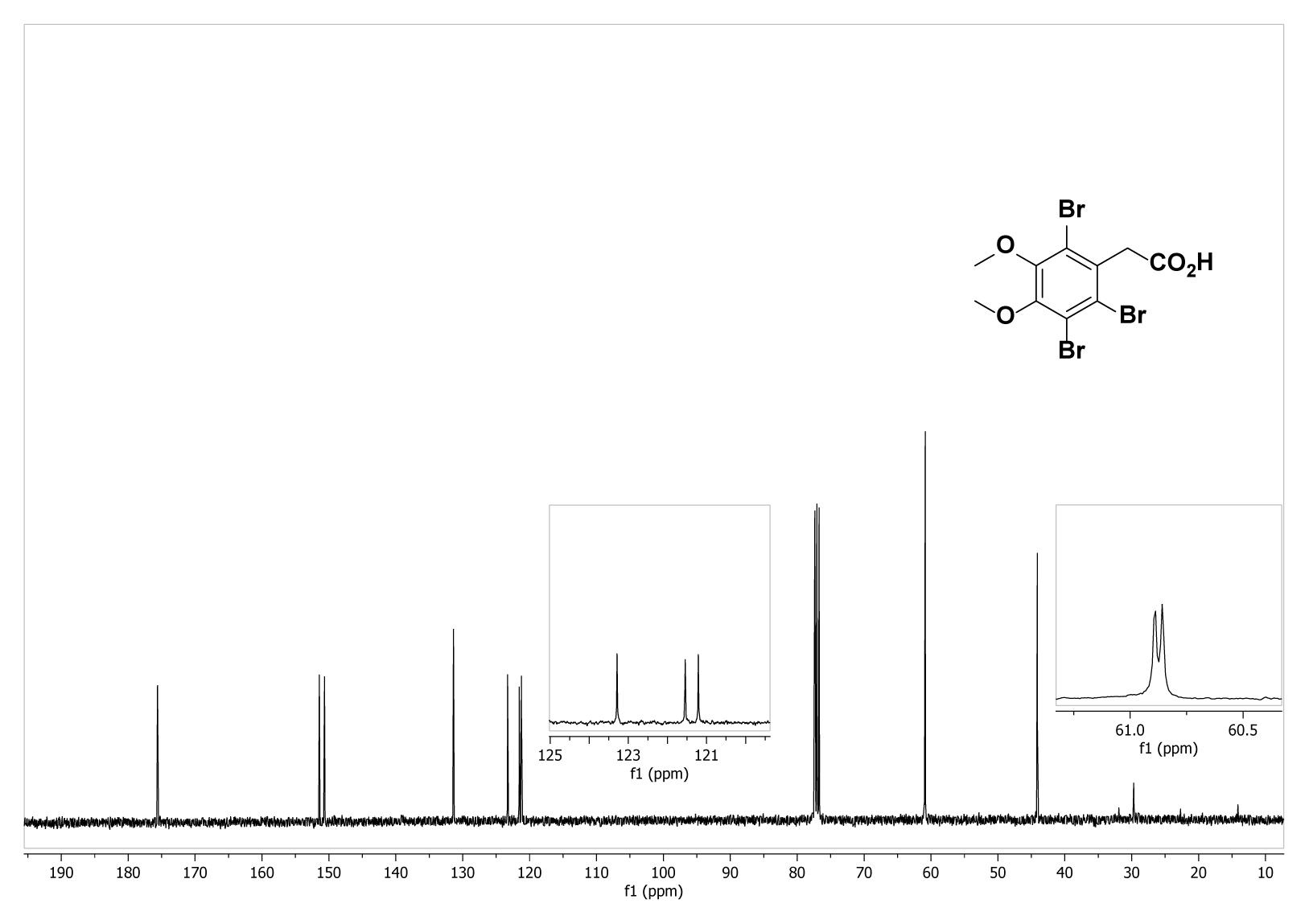

Supplement: Supplementary file 23 — 13C-NMR spectrum of the compound 24 (100 MHz, CDCl3). [file turkjchem-46-5-1405s23.tif]

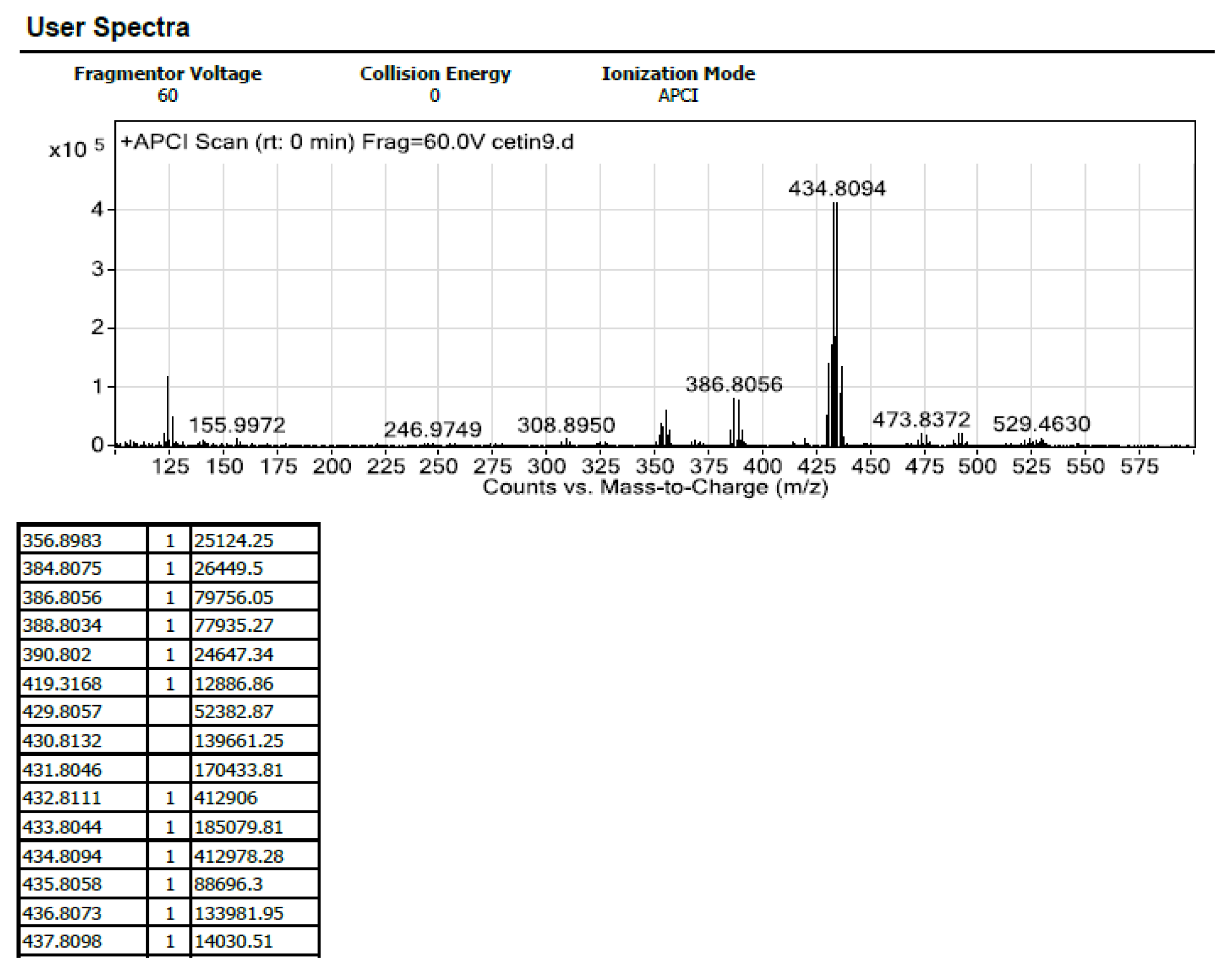

Supplement: Supplementary file 24 — HRMS spectrum of the compound 24. [file turkjchem-46-5-1405s24.tif]

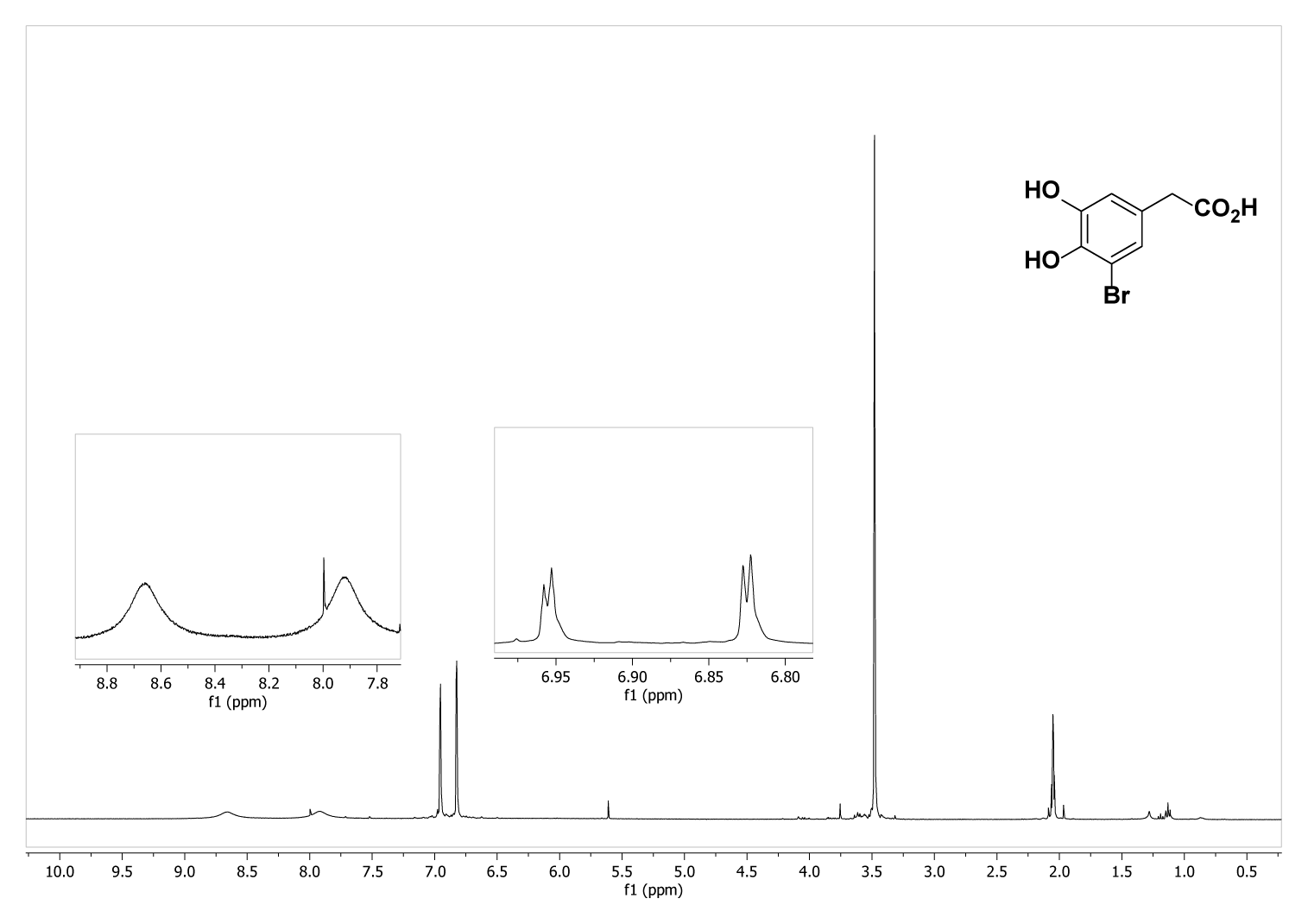

Supplement: Supplementary file 25 — 13C-NMR spectrum of the compound 25 (100 MHz, acetone-d6). [file turkjchem-46-5-1405s25.tif]

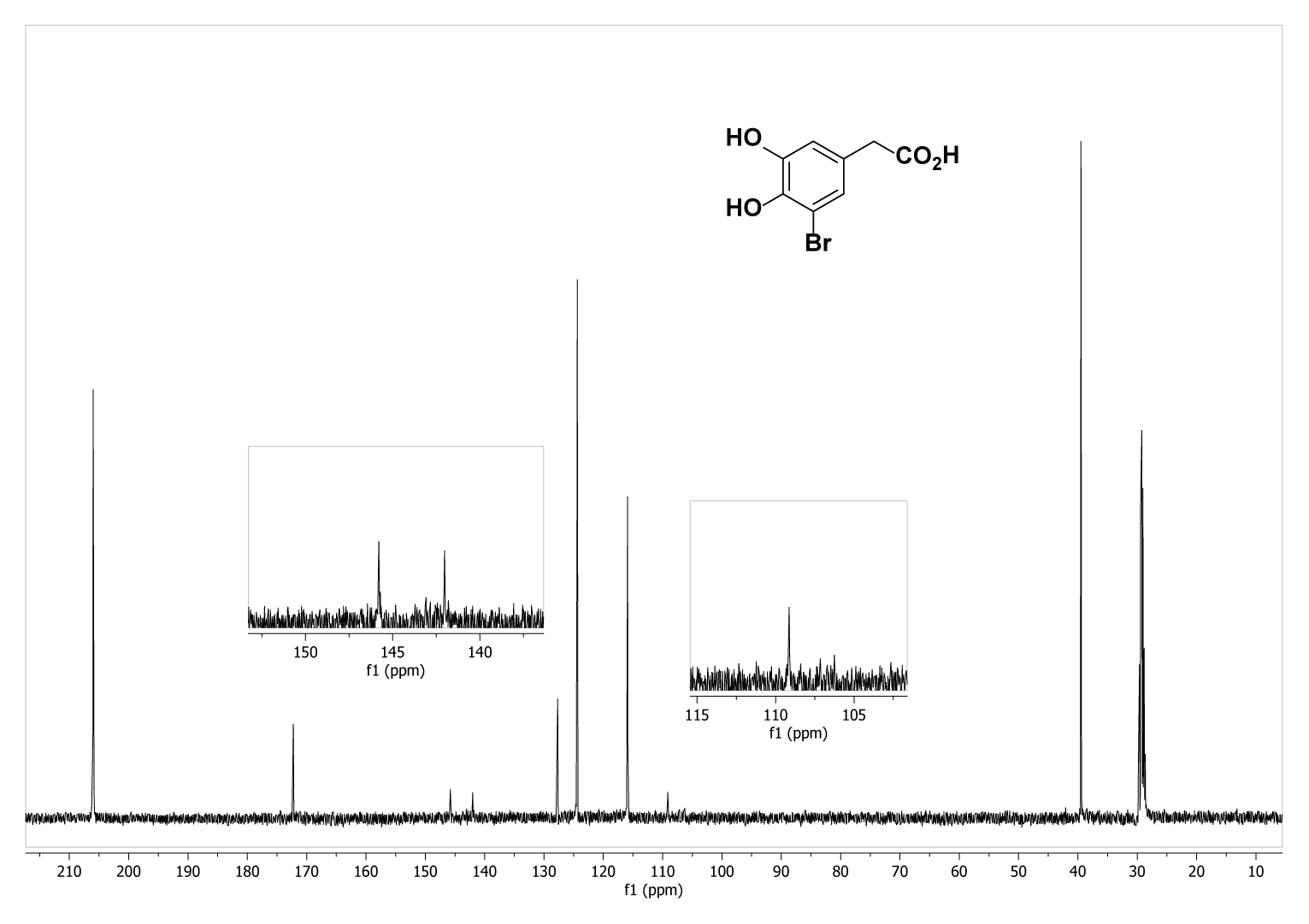

Supplement: Supplementary file 26 — 1H-NMR spectrum of the compound 25 (400 MHz, acetone-d6). [file turkjchem-46-5-1405s26.tif]

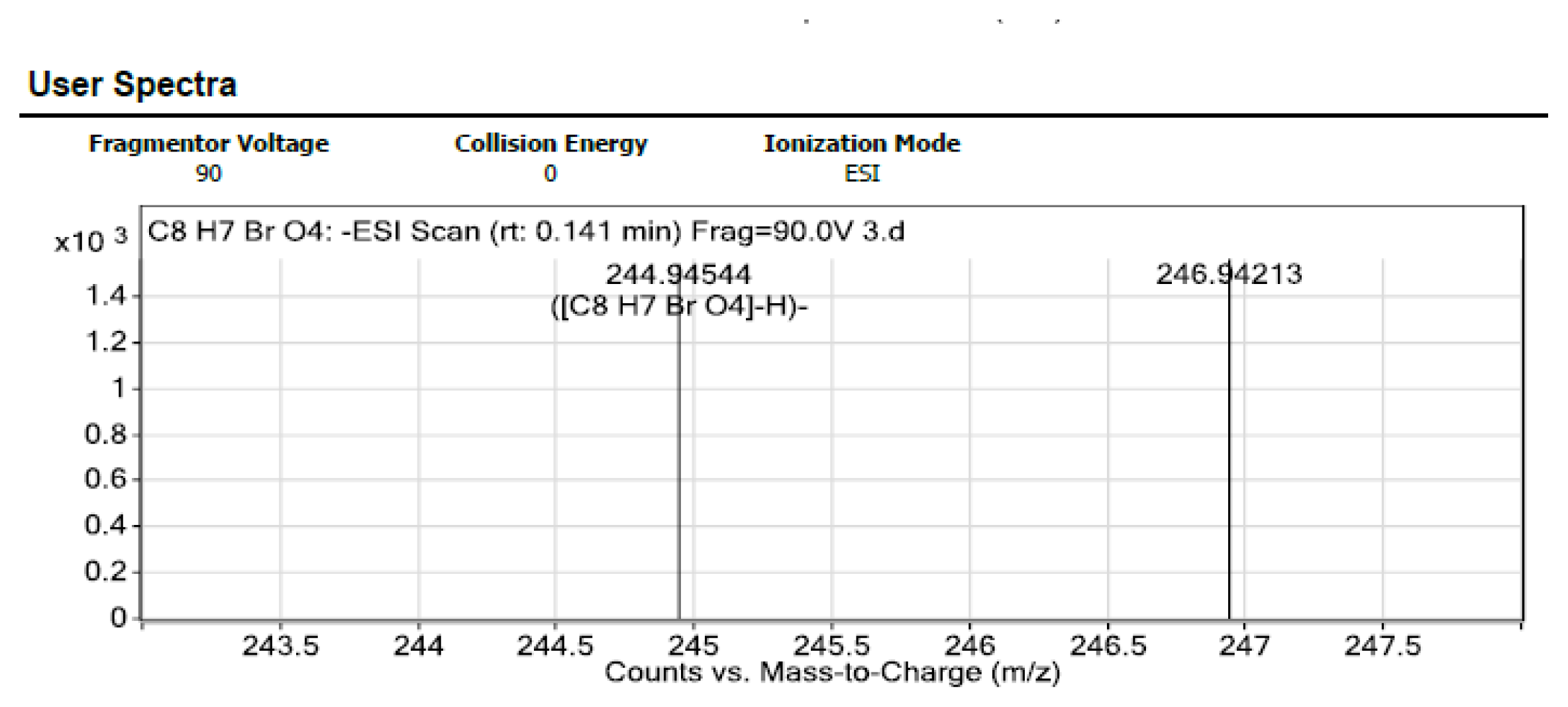

Supplement: Supplementary file 27 — HRMS spectrum of the compound 25. [file turkjchem-46-5-1405s27.tif]

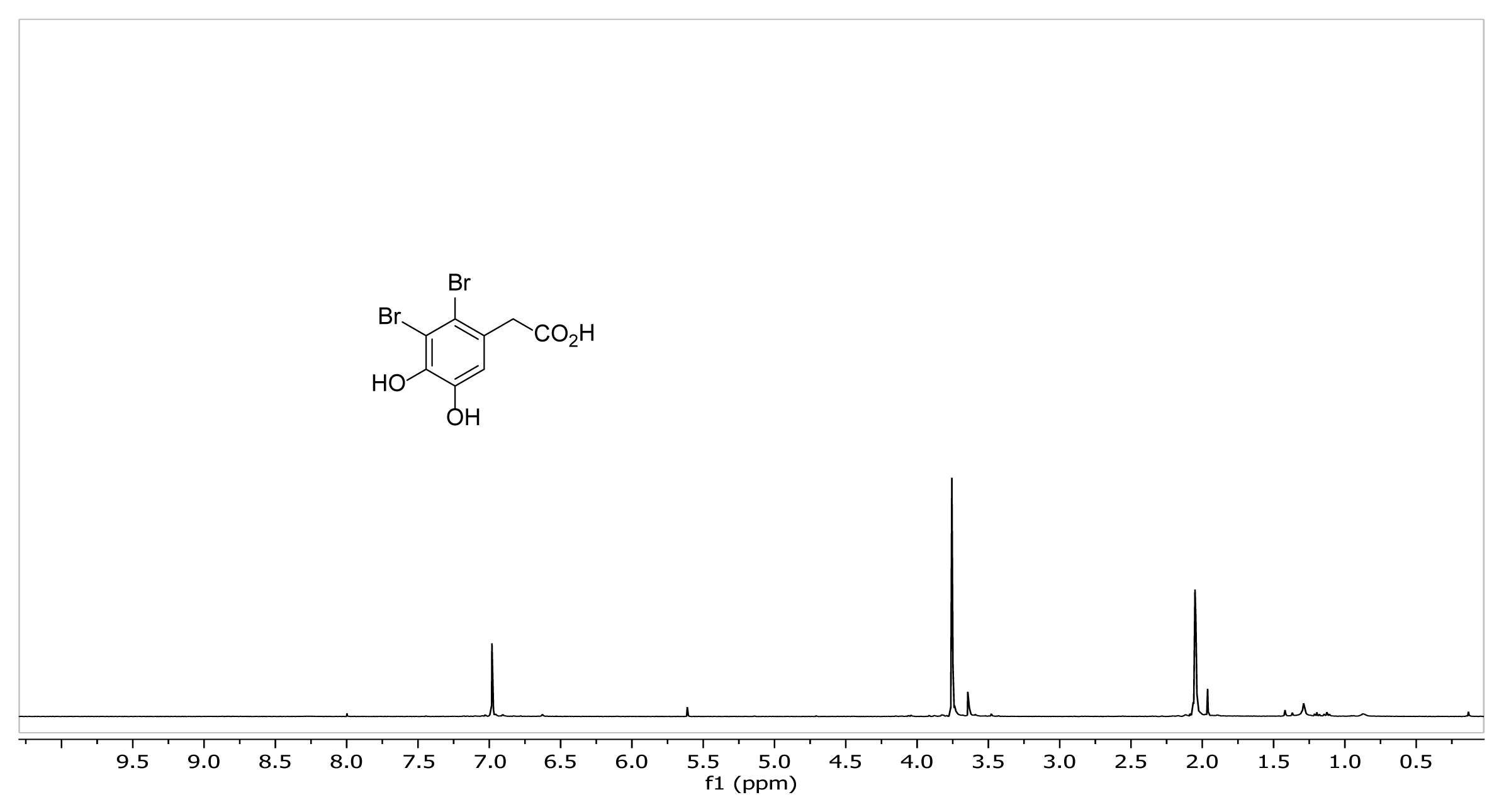

Supplement: Supplementary file 28 — 1H-NMR spectrum of the natural product 1 (400 MHz, acetone-d6). [file turkjchem-46-5-1405s28.tif]

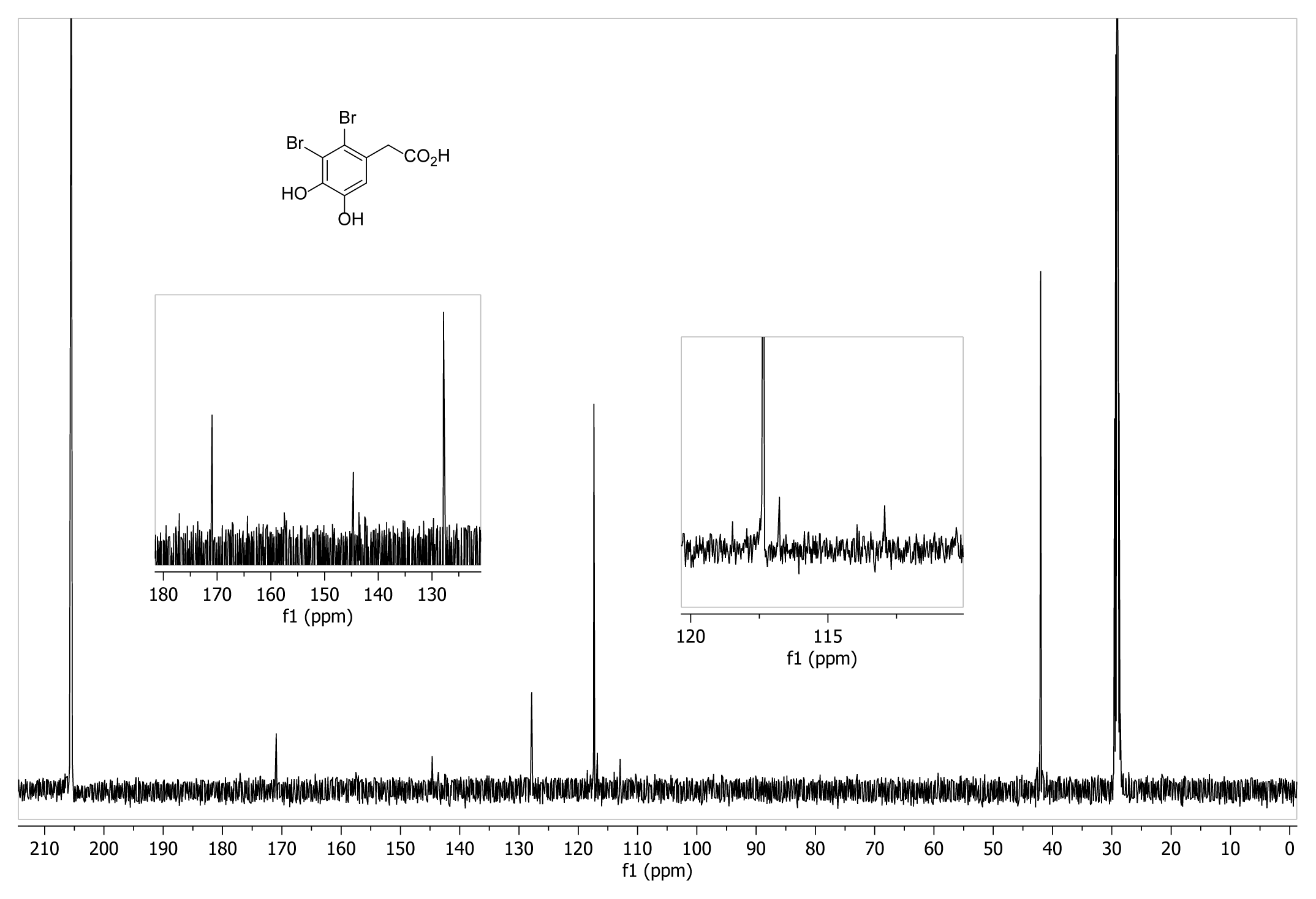

Supplement: Supplementary file 29 — 13C-NMR spectrum of the natural product 1 (100 MHz, acetone-d6). [file turkjchem-46-5-1405s29.tif]

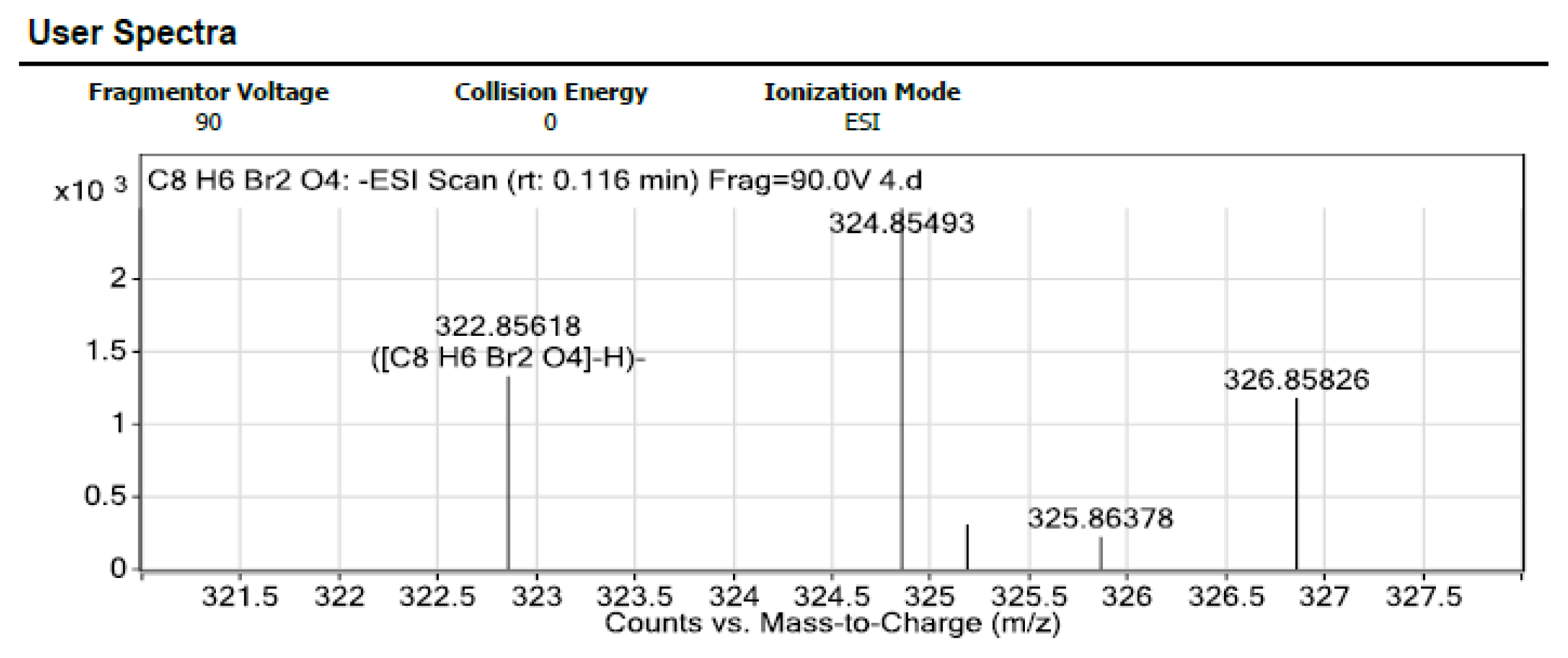

Supplement: Supplementary file 30 — HRMS spectrum of the compound 1. [file turkjchem-46-5-1405s30.tif]

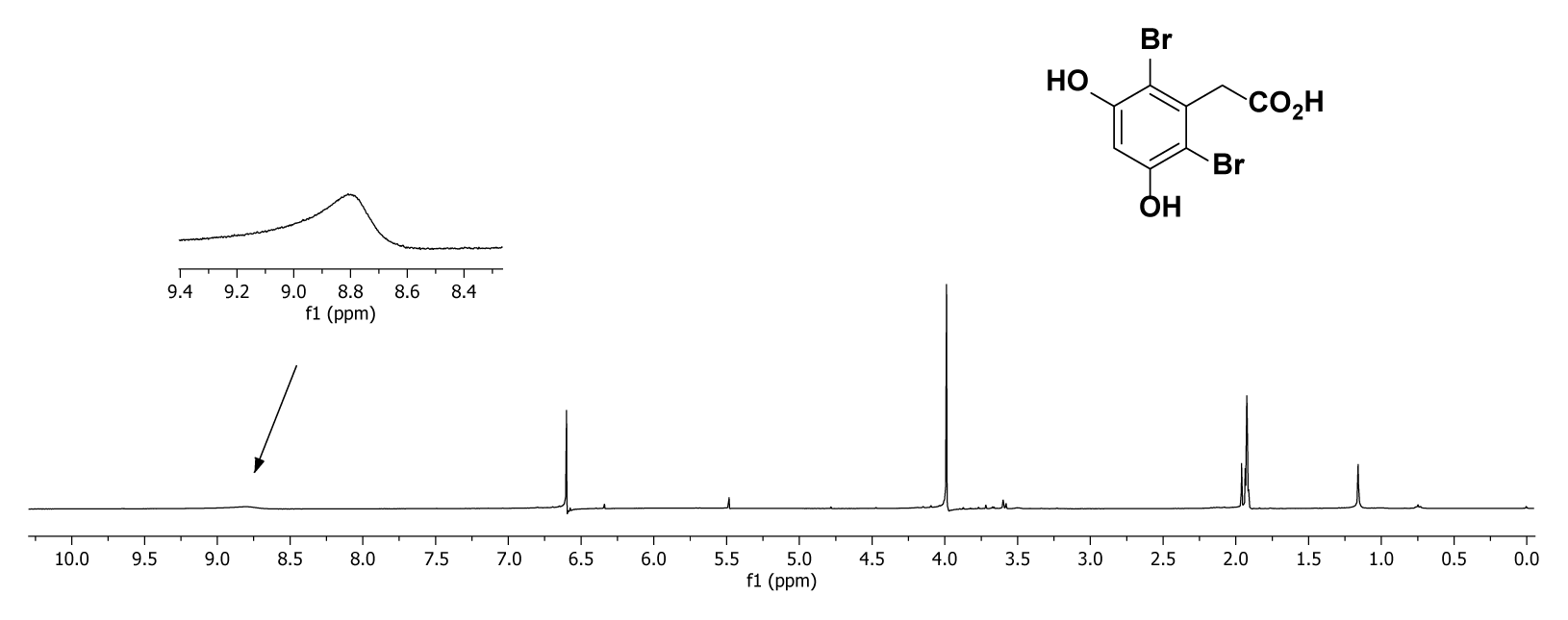

Supplement: Supplementary file 31 — 1H-NMR spectrum of the natural product 2 (400 MHz, acetone-d6). [file turkjchem-46-5-1405s31.tif]

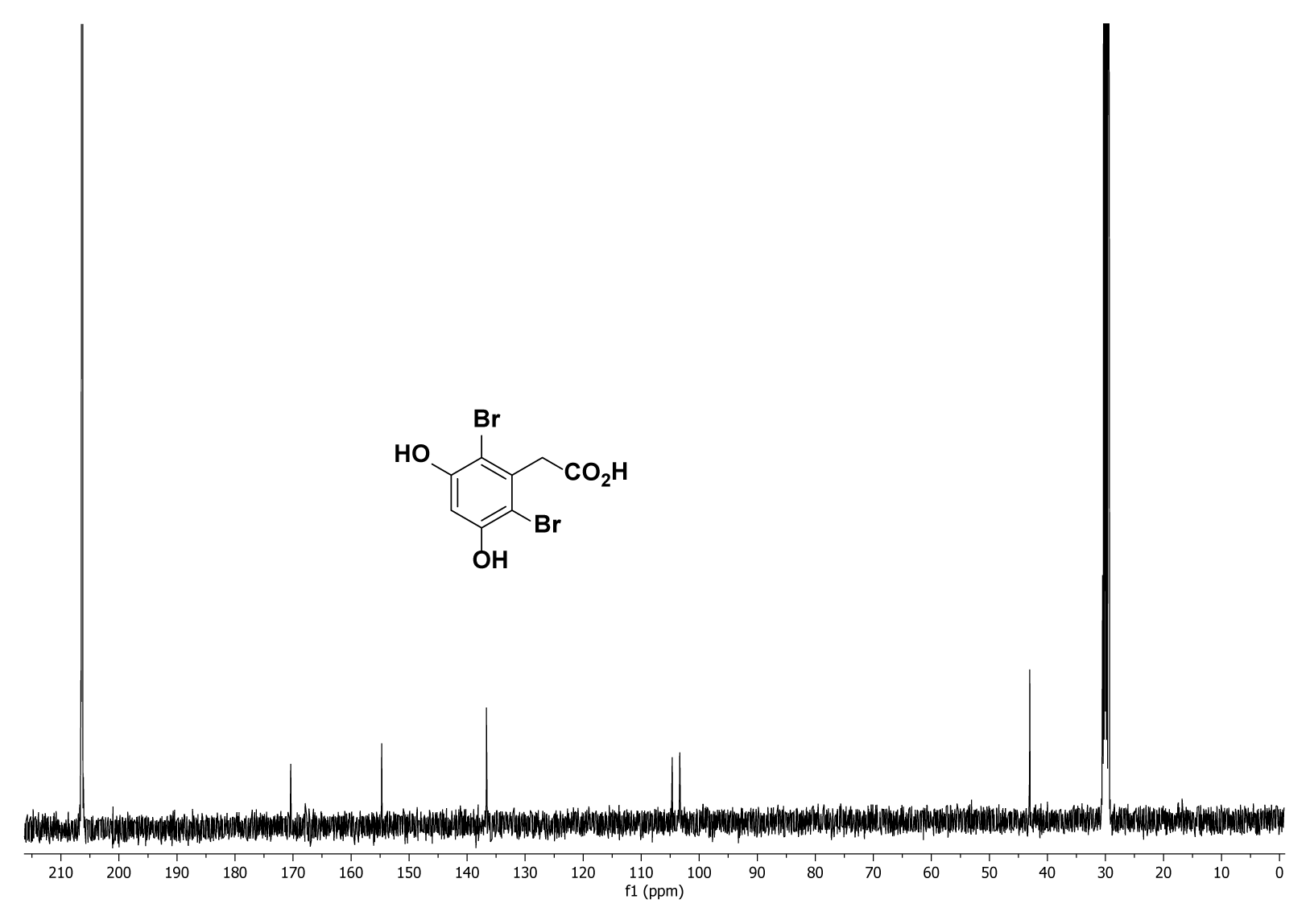

Supplement: Supplementary file 32 — 13C-NMR spectrum of the natural product 2 (100 MHz, acetone-d6). [file turkjchem-46-5-1405s32.tif]

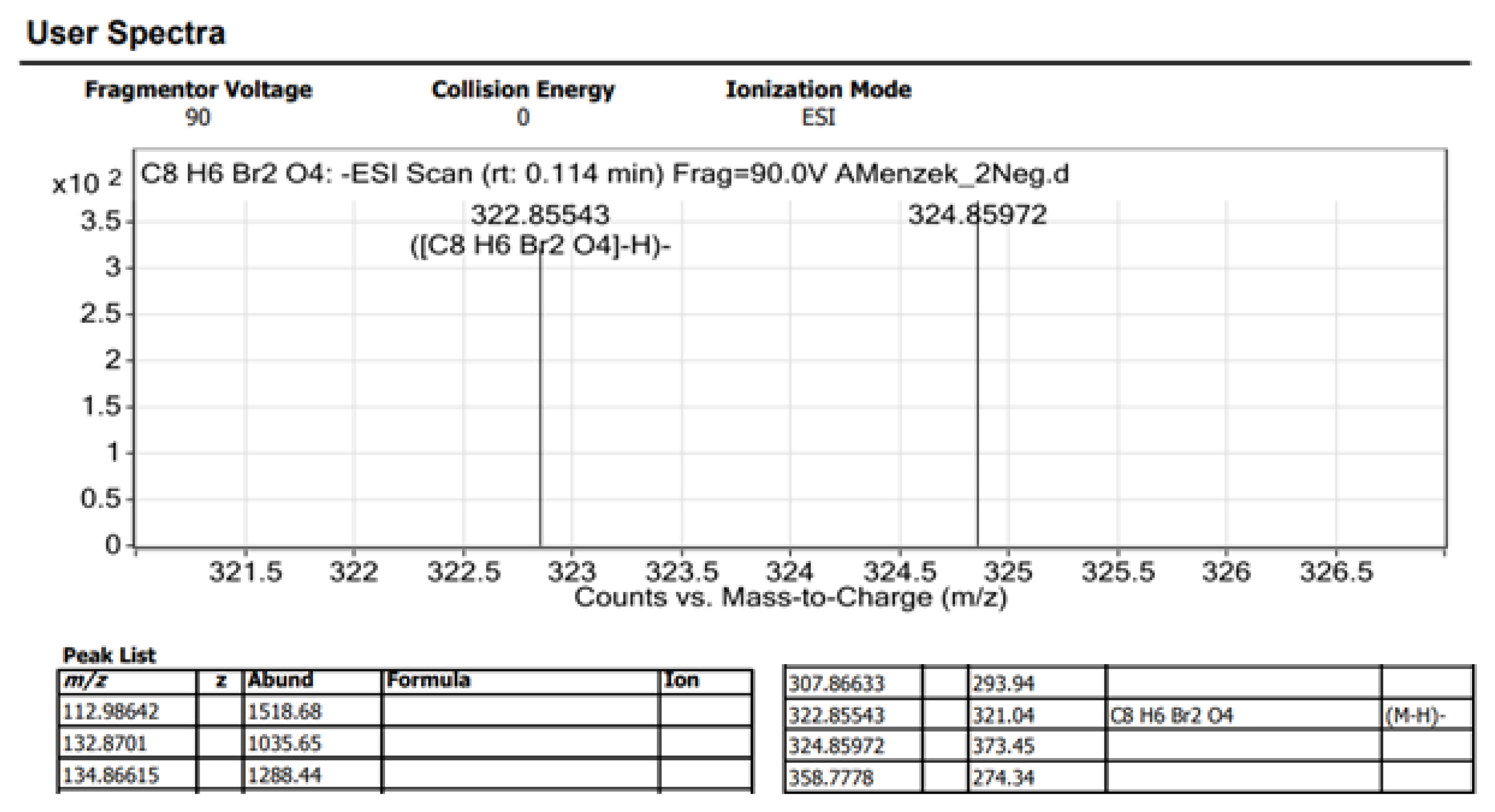

Supplement: Supplementary file 33 — HRMS spectrum of the compound 2. [file turkjchem-46-5-1405s33.tif]

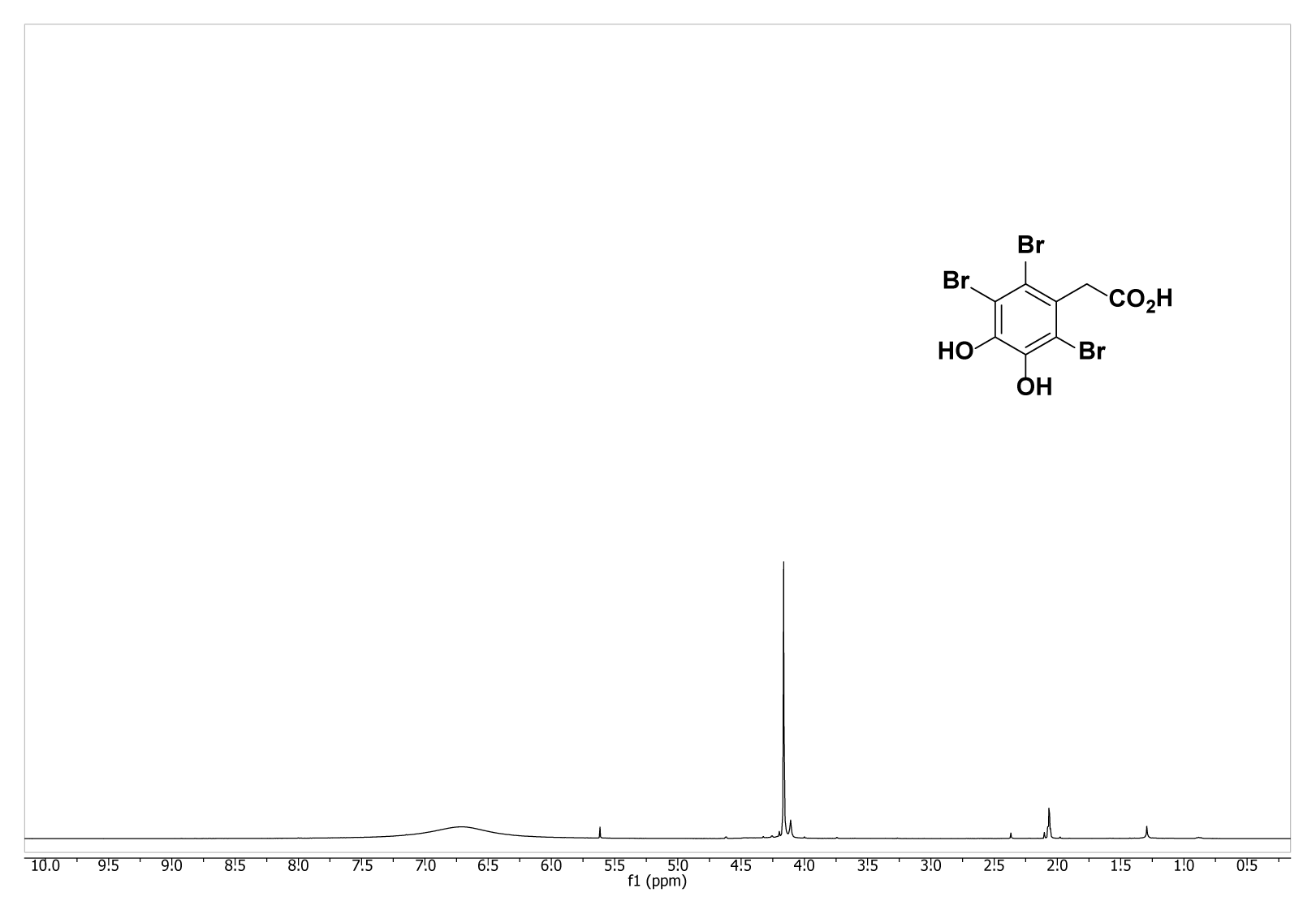

Supplement: Supplementary file 34 — 1H-NMR spectrum of the compound 26 (400 MHz, acetone-d6). [file turkjchem-46-5-1405s34.tif]

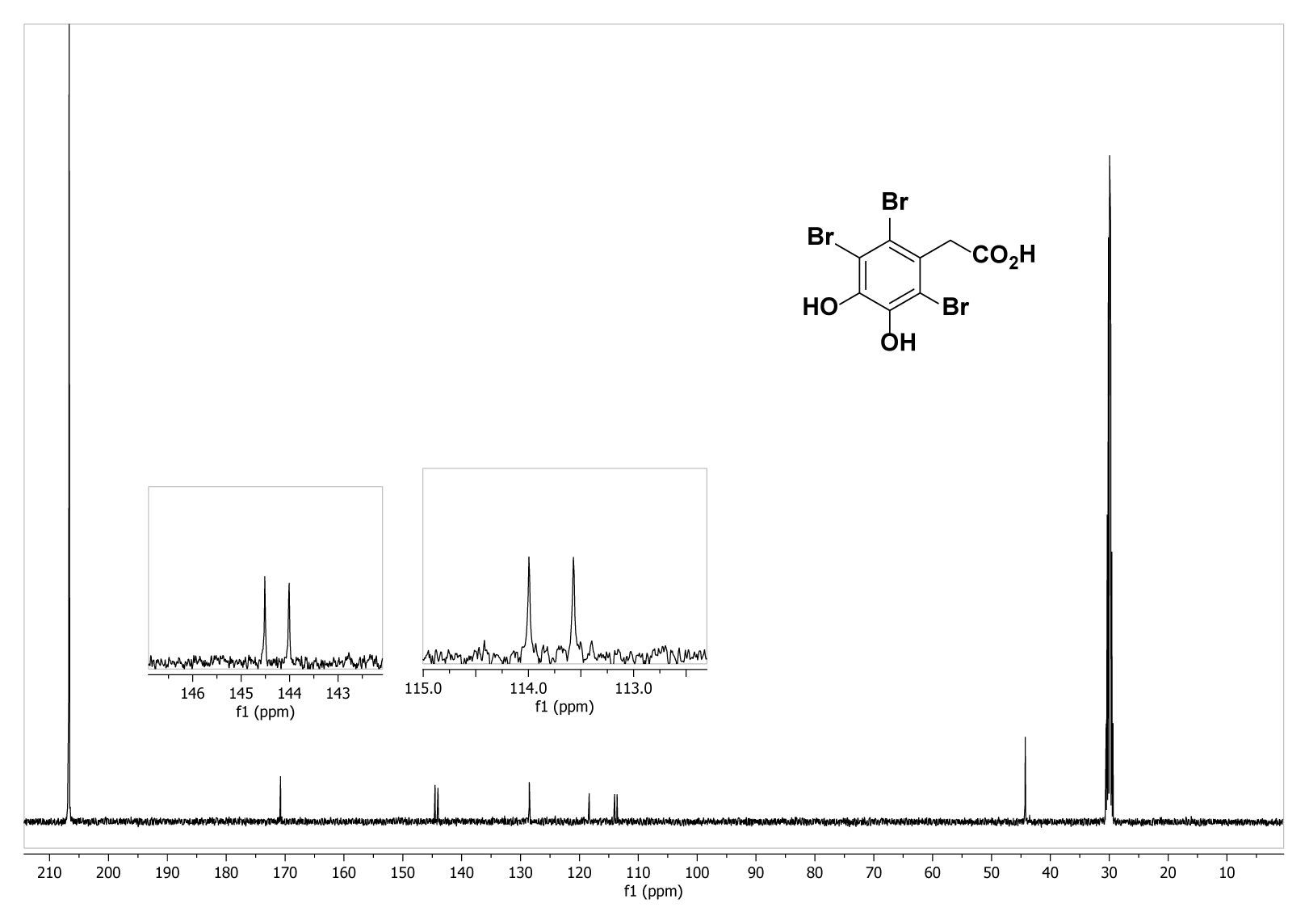

Supplement: Supplementary file 35 — 13C-NMR spectrum of the compound 26 (100 MHz, acetone-d6). [file turkjchem-46-5-1405s35.tif]

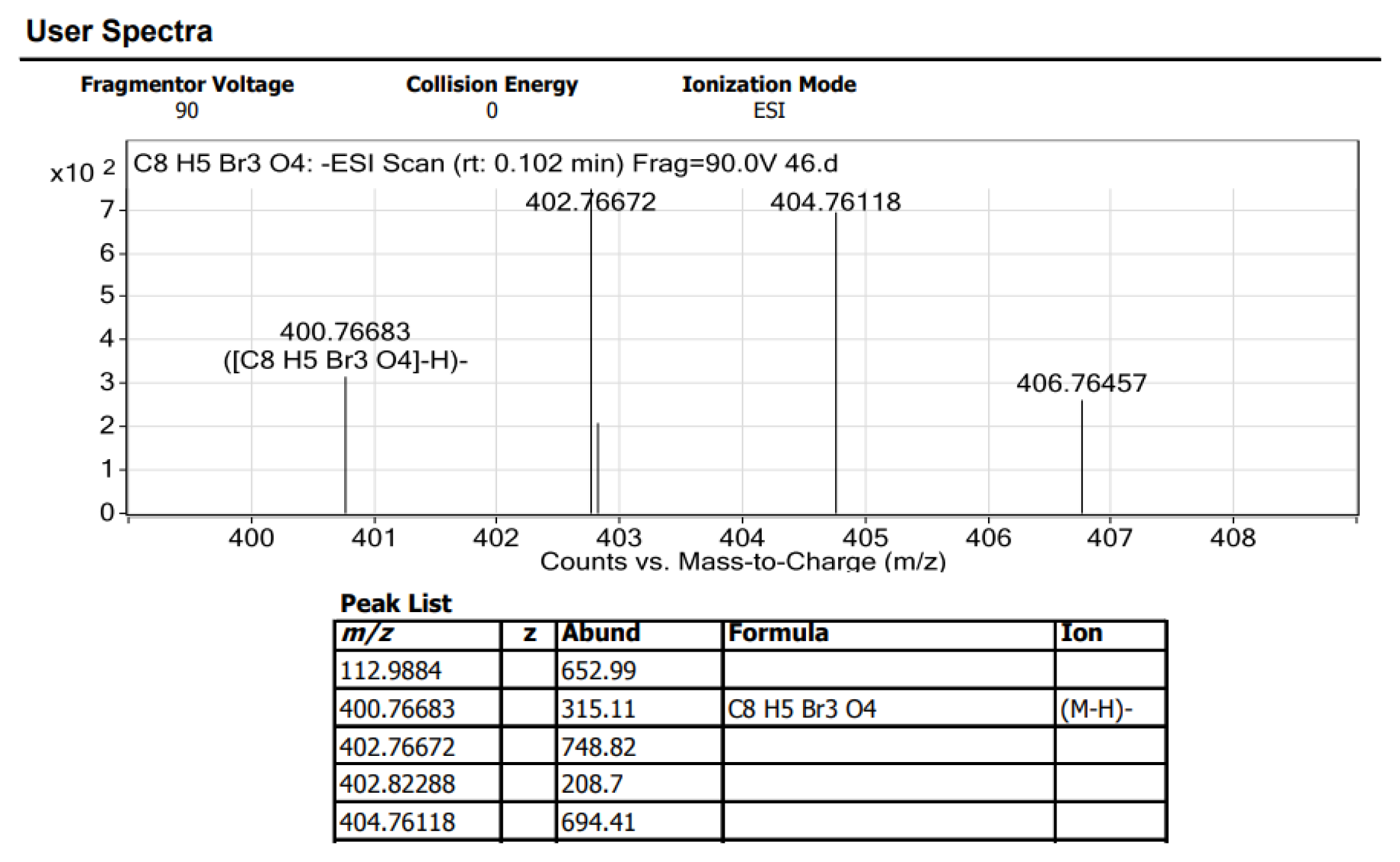

Supplement: Supplementary file 36 — HRMS spectrum of the compound 26. [file turkjchem-46-5-1405s36.tif]

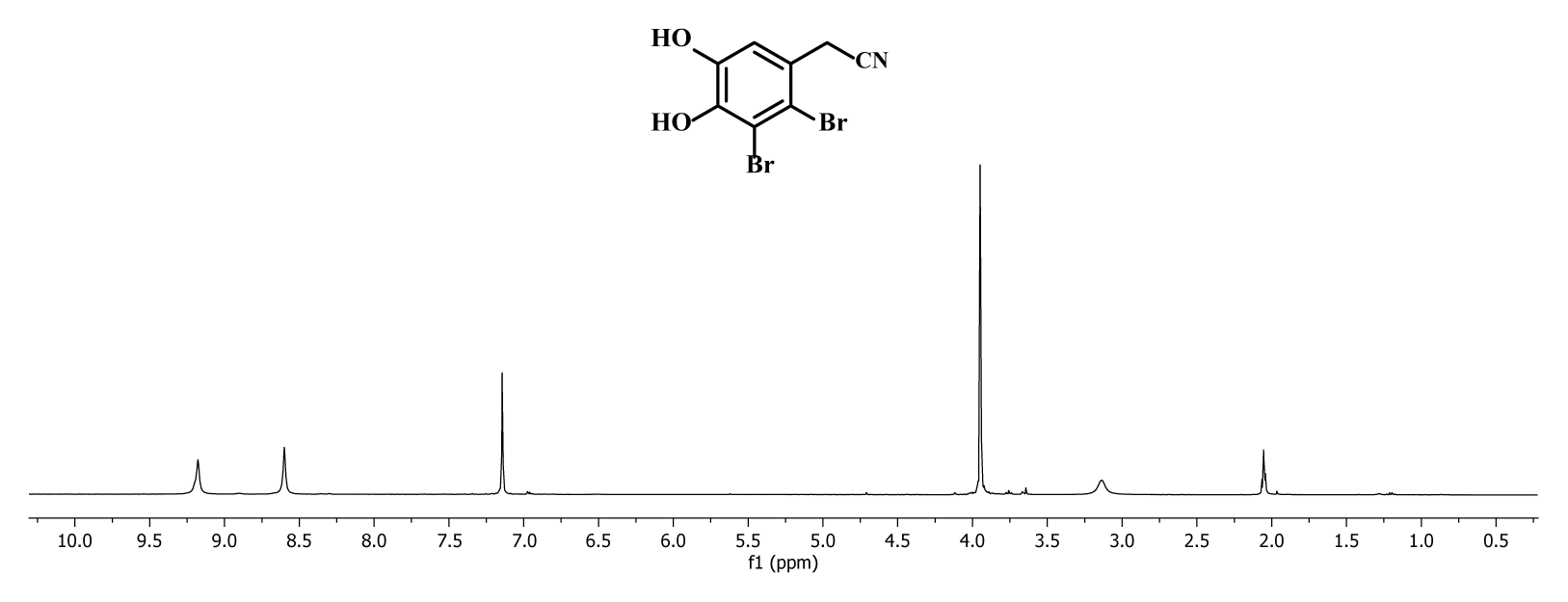

Supplement: Supplementary file 37 — 1H-NMR spectrum of the compound 27 (400 MHz, acetone-d6). [file turkjchem-46-5-1405s37.tif]

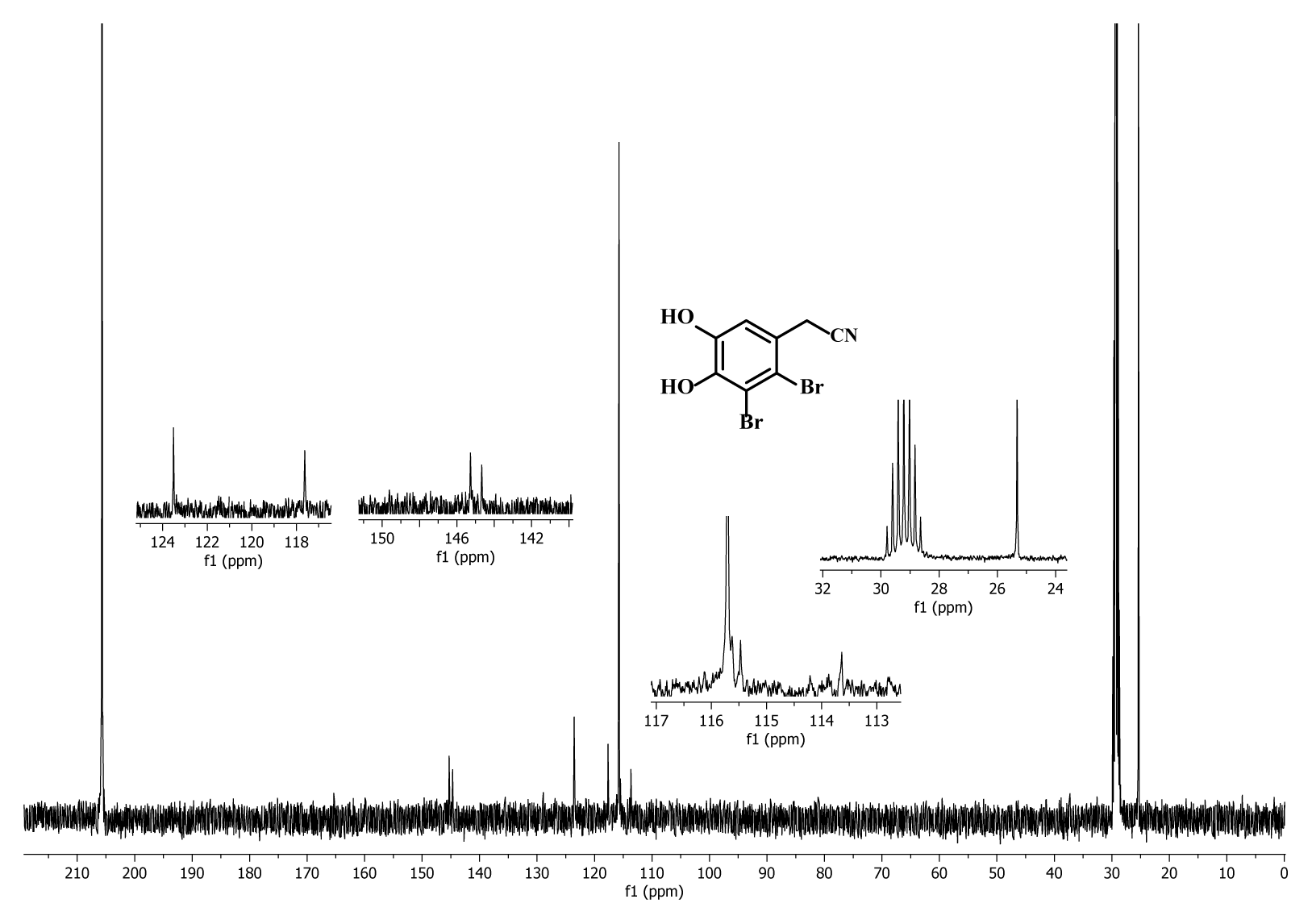

Supplement: Supplementary file 38 — 13C-NMR spectrum of the compound 27 (100 MHz, acetone-d6). [file turkjchem-46-5-1405s38.tif]

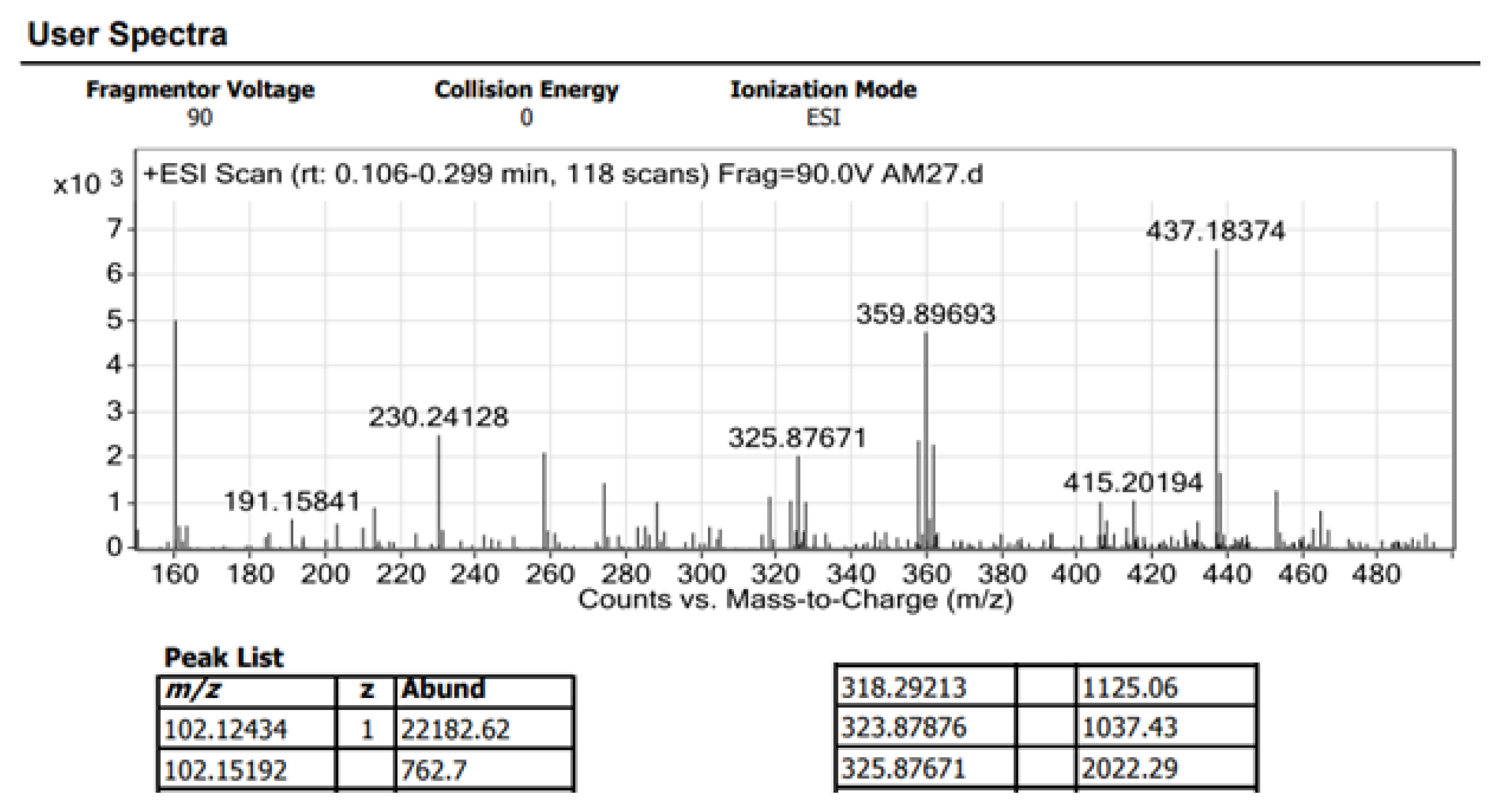

Supplement: Supplementary file 39 — HRMS spectrum of the compound 27. [file turkjchem-46-5-1405s39.tif]

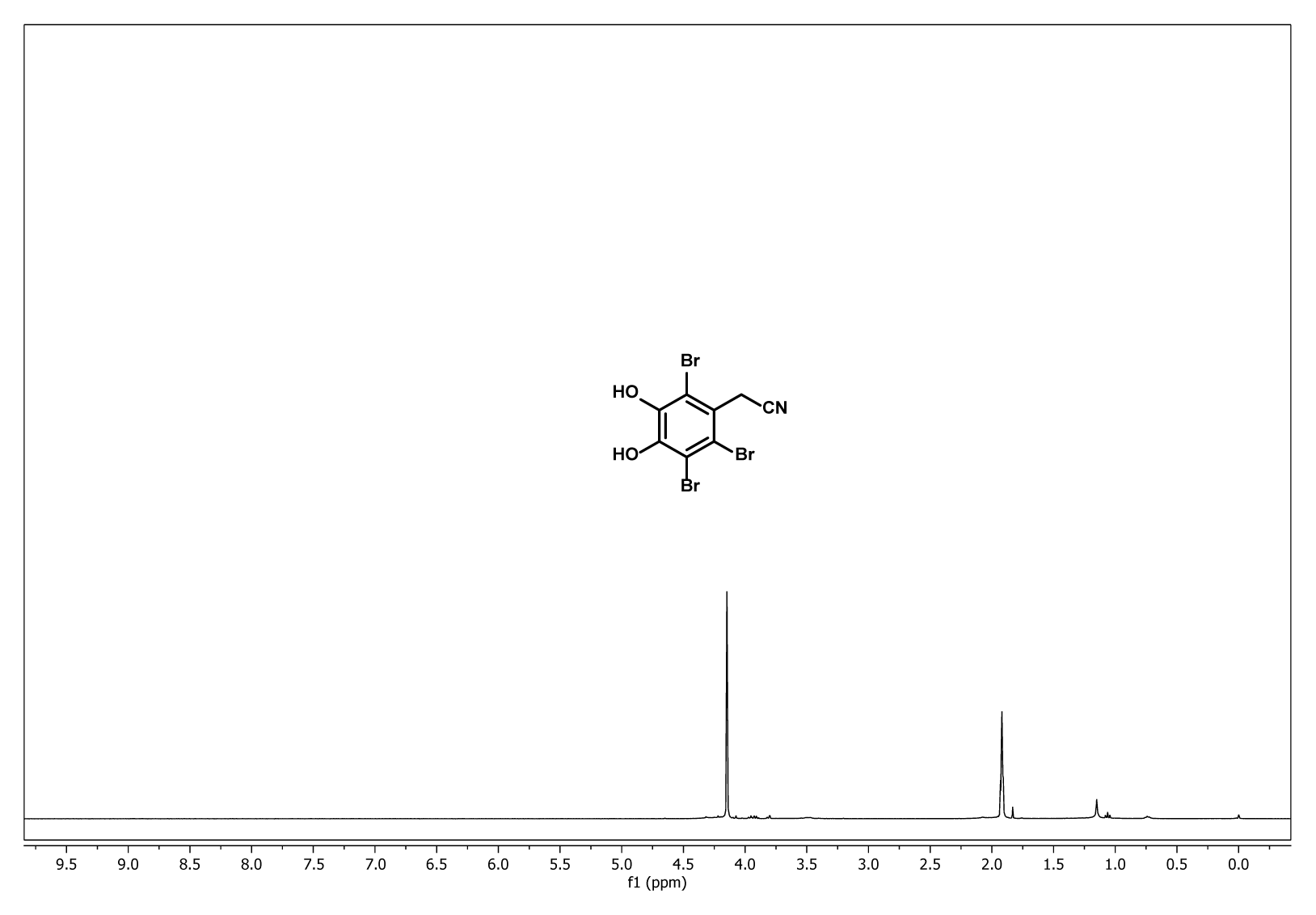

Supplement: Supplementary file 40 — 1H-NMR spectrum of the compound 28 (400 MHz, acetone-d6). [file turkjchem-46-5-1405s40.tif]

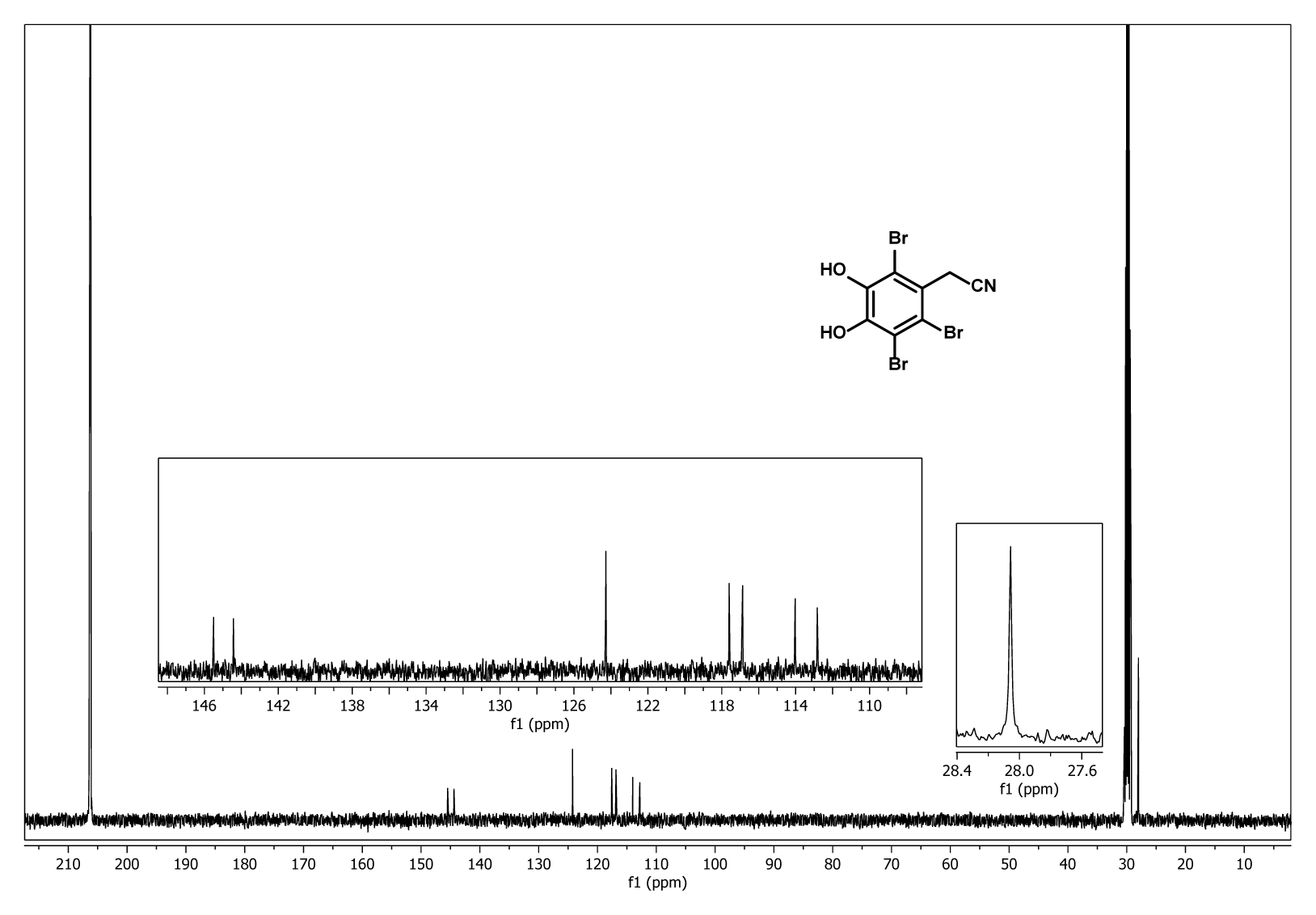

Supplement: Supplementary file 41 — 13C-NMR spectrum of the compound 28 (100 MHz, acetone-d6). [file turkjchem-46-5-1405s41.tif]

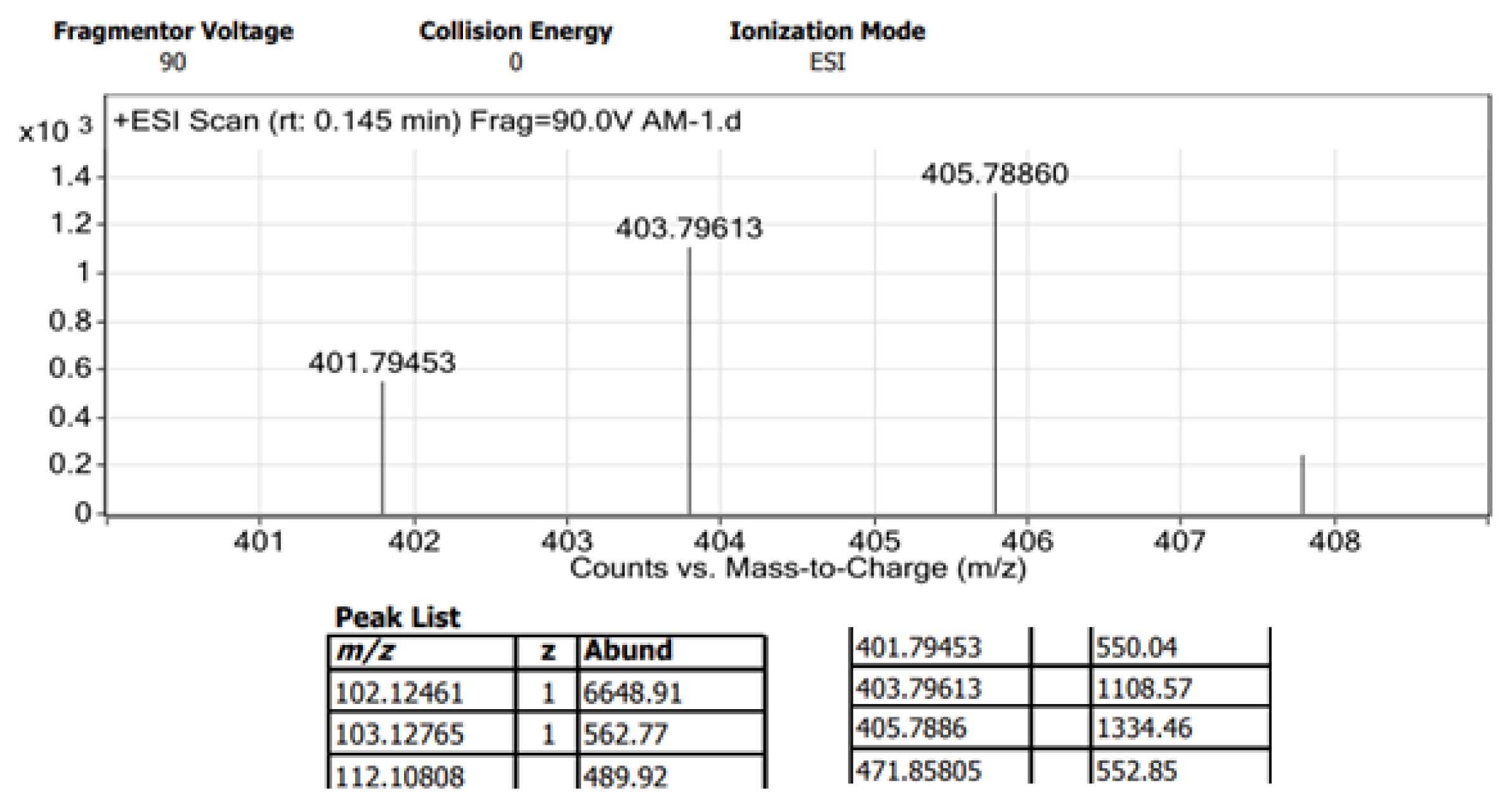

Supplement: Supplementary file 42 — HRMS spectrum of the compound 28. [file turkjchem-46-5-1405s42.tif]
